# Regulation of density of functional presynaptic terminals by local energy supply

**DOI:** 10.1186/s13041-015-0132-z

**Published:** 2015-07-17

**Authors:** Hang Zhou, Guosong Liu

**Affiliations:** Department of Basic Medical Sciences, School of Medicine, Tsinghua University, Beijing, China

**Keywords:** Density of functional presynaptic terminals, Energy supply, Presynaptic Ca^2+^ sensitivity, Ca^2+^-sensitivity-related proteins, Axonal transport, Intracellular Mg^2+^

## Abstract

**Background:**

The density of functional synapses is an important parameter in determining the efficacy of synaptic transmission. However, how functional presynaptic terminal density is regulated under natural physiological conditions is still poorly understood.

**Results:**

We studied the factors controlling the density of presynaptic functional terminals at single dendritic branches of hippocampal neurons and found that elevation of intracellular Mg^2+^ concentration was effective in increasing the density of functional terminals. Interestingly, the upregulation was not due to synaptogenesis, but to the conversion of a considerable proportion of presynaptic terminals from nonfunctional to functional. Mechanistic studies revealed that the nonfunctional terminals had inadequate Ca^2+^-sensitivity-related proteins, resulting in very low Ca^2+^ sensitivity within their vesicle release machinery. We identified energy-dependent axonal transport as a primary factor controlling the amount of Ca^2+^-sensitivity-related proteins in terminals. The elevation of intracellular Mg^2+^ enhanced local energy supply and promoted the increase of Ca^2+^-sensitivity-related proteins in terminals, leading to increased functional terminal density.

**Conclusions:**

Our study suggests that local energy supply plays a critical role in controlling the density of functional presynaptic terminals, demonstrating the link between energy supply and efficacy of synaptic transmission.

**Electronic supplementary material:**

The online version of this article (doi:10.1186/s13041-015-0132-z) contains supplementary material, which is available to authorized users.

## Background

Functional synapses are the elemental unit of synaptic computation in the neural network, and the density of functional synapses determines the capacity for information transmission [1]. Interestingly, under physiological conditions, a considerable number of synapses are nonfunctional (i.e. silent/dormant), and the ratio of functional/nonfunctional synapses fluctuates over time. These fluctuations have important implications in brain functions [2], including in neural development [3], physiological function maintenance [4–7], multiple neural pathologies [8, 5, 9–11] and drug addictions [12].

Generally, synaptic silence can be divided into two categories based on the locus of silence, either pre- or postsynaptic. Molecular mechanisms of postsynaptic silence have been studied extensively, determining that lack of AMPA receptors at the postsynaptic locus is largely responsible for postsynaptic silence [13, 14, 5, 15, 2]. However, the mechanisms of presynaptic silence are still elusive [16, 17].

The presence of nonfunctional presynaptic release sites was first theorized from early quantal analysis studies, and later directly observed with the application of FM dyes, showing a considerable number of nonfunctional presynaptic terminals (with extremely low release probability, *Pr*) [18–21]. A large amount of research has been done to understand the mechanisms underlying the presence of nonfunctional terminals. Convergence of evidence suggests that presynaptic silence can be regulated by perturbations in network excitability. For instance, presynaptic terminals can be silenced by chronic depolarization [22, 23] or glutamate excitotoxicity [9]. Or, nonfunctional terminals can be activated by chronic (in several hours) blockage of action potentials [23, 24], but surprisingly the activated terminals will be silenced again if the blockage is prolonged (>48 hr) [25].

Multiple signaling pathways are involved in presynaptic nonfunctional-functional switching [26], such as the activation of presynaptic Ca^2+^ related pathways [27], cAMP/PKA related pathways [21, 28, 29] and diacylglycerol related pathways [30–32], and the inhibition of CDK5 pathways [33–35]. Other mechanisms thought to be involved in presynaptic nonfunctional-functional switching include retrograde signaling [36–40] and the activation of presynaptic proteasome degradation [41, 42, 25, 43]. Presynaptic cytomatrix proteins (e.g. RIM1, Munc13, ELKS etc.) [26, 16], which are critical for vesicle turnover [44], seem to be the targets of these regulations. For example, Munc13 mediates the increase in transmitter release induced by administration of phorbal ester or diacylglycerol [45].

While there is a plethora of information regarding the mechanisms involved in presynaptic nonfunctional-functional switching, there are still major unsolved issues. (1) Is the presynaptic nonfunction (silence) a normal physiological state of presynaptic terminals [16]? If so, what are the physiological parameters that determine the ratio of functional/nonfunctional terminals, and in turn, determine the functional synapse density at dendrites? Most of the previous studies used extreme treatments, such as high K^+^, high concentration of ambient glutamate, intense AP stimulation for hours and complete inhibition of APs for days [9, 22–25, 42]. Studies using these treatments can help understand the regulation of nonfunctional-functional conversion under pathological processes, but still might not provide accurate information on regulation of nonfunctional terminals under natural physiological conditions. (2) What molecular factors are necessary in presynaptic terminals to make them functional under physiological conditions? One strategy to answer this question is to delete presynaptic proteins and look at the loss of presynaptic functions [44]. However, we are more interested in what presynaptic proteins are “missing” in natural state. If the presence of nonfunctional presynaptic terminals is an integral part of physiological regulation of synaptic strength, then identification of the “missing” proteins in presynaptic nonfunctional terminals will help elucidate the molecular mechanisms controlling the presynaptic nonfunctional-functional switching under physiological conditions. Furthermore, modifying the quantity of the identified proteins might serve as a way to regulate the density of functional synapses.

In the current study, we applied bursting action potentials (correlated activity) as input and quantified the functional status of presynaptic terminals in cultured hippocampal neuronal networks by their ability to undergo vesicle turnover. We found in this study that elevating extracellular Mg^2+^ concentration results in an increase in intracellular Mg^2+^ and subsequently a conversion of a majority of the nonfunctional terminals to functional status. Therefore, we manipulated extracellular Mg^2+^ as a tool to help determine the mechanisms involved in this process, including presynaptic Ca^2+^ sensitivity, Ca^2+^-sensitivity-related protein turnover and energy supply. In conclusion, our study suggests that local energy supply is an important physiological factor that regulates the ratio of functional/nonfunctional terminals, achieving a regulation of the density of functional terminals. Moreover the concentration of intracellular Mg^2+^ might serve as a messenger for such regulation of functional terminal density.

## Results

### Functional terminal density is determined by intracellular Mg^2+^

**Fig. 1:**
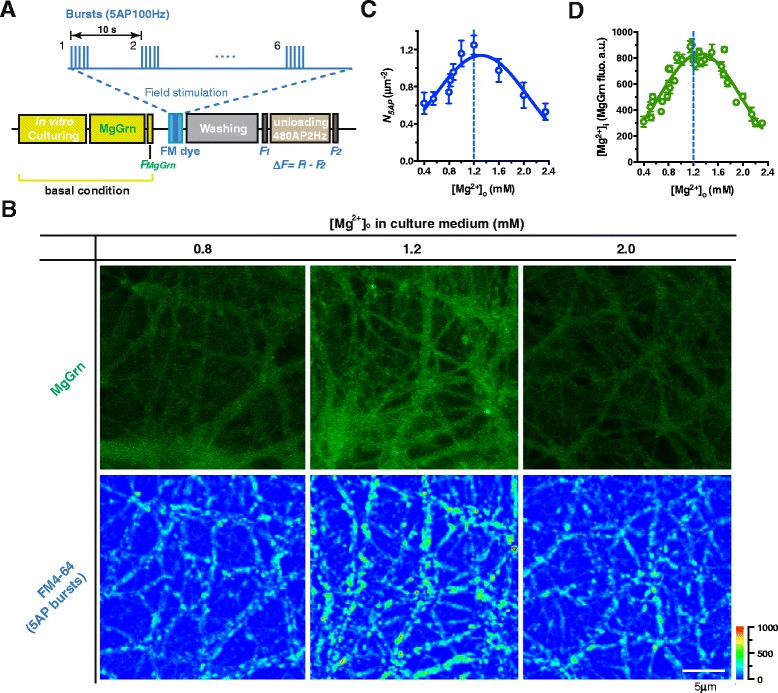
Functional terminal density in response to 5AP bursts is nonlinearly associated with extracellular Mg^2+^ concentration. (**a**) The schematic experimental procedures. Magnesium Green-AM ester (MgGrn) staining and imaging were performed at basal condition (close to natural culturing state, without eliciting any stimulus). After MgGrn imaging, vesicle turnover was detected by FM dye under field stimulations, such as bursting stimulations (e.g. 5AP bursts). For the bursting stimulation, 30 action potentials (APs) were divided into 6 bursts (inter-burst-interval was 10 s), each of which contained 5 APs at 100 Hz. The MgGrn and FM dye imaging procedures were combined (e.g. for the experiment in **b**) or conducted separately based on different experimental designs. (**b**) Neuron cultures with [Mg^2+^]_o_ of 0.8, 1.2 or 2.0 mM in culture medium (for 48 hr to 2 weeks) were marked by MgGrn to reveal their [Mg^2+^]_i_ level, and then functional terminals were detected by FM4-64 under 5AP bursts (as described in **a**) at the same area of interests (AOIs). Pseudo-color scale: fluorescent intensity. (**c**) Bell-shape association between functional terminal density in response to 5AP bursts (*N*_*5AP*_) and [Mg^2+^]_o_ (n = 3–5 coverslips, Gaussian curve fitting, R^2^ = 0.84). (**d**) Bell-shape association between [Mg^2+^]_i_ (MgGrn fluorescence) and [Mg^2+^]_o_ (n = 3–5 coverslips, Gaussian curve fitting, R^2^ = 0.86). The mean ± SEM of coverslips was presented. For the measurement of *N*_*5AP*_ and [Mg^2+^]_i_ see Methods

In the current study, we used bursting action potentials (APs) (correlated activity) as input to quantify the functional synapse density. Bursting APs were chosen because they play an essential role in information transmission in both the developmental and mature networks in the hippocampus [46–49]. Because bursts in the hippocampus usually contain 2–6 high-frequency (>100Hz) APs under physiological conditions [50], and a special form of bursts, the “theta bursts” (usually 4–5 APs in a burst), is found effective at inducing long-term plasticity [51, 52], here we chose 5AP bursts as input stimuli for quantification of the density of functional terminals. The stimuli sequence contained 6 groups of 5AP bursts with an inter-burst-interval of 10 s (30 APs total) (Fig. 1a). FM1-43 or FM4-64 was used to detect vesicle turnover of functional terminals (Fig. 1a) [24]. Here, a terminal was defined as functional if releasable FM dye was detectable following bursting input stimulation. A terminal was considered to be nonfunctional if it failed to release even one vesicle after 30 AP bursting input (i.e. no detectable FM dye; *Pr* < 0.04).

Our prior work shows that elevating extracellular Mg^2+^ concentration ([Mg^2+^]_o_) from 0.8 to 1.2 mM can efficiently enhance presynaptic plasticity [24]. Here we studied whether the change of [Mg^2+^]_o_ could influence functional terminal density. After a long term (48 hr to 2 weeks) increase of [Mg^2+^]_o_ in culture medium, we found that functional terminal density (*N*_*5AP*_) was positively proportional to [Mg^2+^]_o_ as [Mg^2+^]_o_ increased from 0.4 to 1.2 mM. However, to our surprise, *N*_*5AP*_ decreased as [Mg^2+^]_o_ further increased from 1.2 to 2.4 mM (Fig. 1b lower and c). To understand why *N*_*5AP*_ and [Mg^2+^]_o_ exhibited such a bell-shape relationship, we investigated the relationship between [Mg^2+^]_o_ and intracellular Mg^2+^ concentration ([Mg^2+^]_i_), since intracellular Mg^2+^ is conventionally considered an important endogenous factor involved in multiple intracellular regulations. We used an intracellular Mg^2+^ indicator, Magnesium Green-AM ester (MgGrn), to label intracellular Mg^2+^ at basal condition (without eliciting any AP stimulus), the fluorescent intensity of MgGrn could be considered proportional to [Mg^2+^]_i_ (see Methods). Thus we used MgGrn fluorescence to determine the relative quantity of [Mg^2+^]_i_ in each branch and to estimate the average [Mg^2+^]_i_ at a local area of the network (see Methods). By measuring MgGrn fluorescence in the neurons cultured in different [Mg^2+^]_o_ culture media (for 48 hr to 2 weeks), we found that altering [Mg^2+^]_o_ led to a dramatic change in MgGrn fluorescence at basal condition (Fig. 1b upper; the MgGrn images were taken before FM staining at the same area of the network, for procedures see Fig. 1a). Interestingly, [Mg^2+^]_i_ (represented by the corrected MgGrn fluorescence, see Methods) versus [Mg^2+^]_o_ exhibited a bell-shape relationship (Fig. 1d) similar to that of *N*_*5AP*_ versus [Mg^2+^]_o_ (Fig. 1c). These data implied the possibility that *N*_*5AP*_ was closely correlated with [Mg^2+^]_i_ but not [Mg^2+^]_o_.

**Fig. 2:**
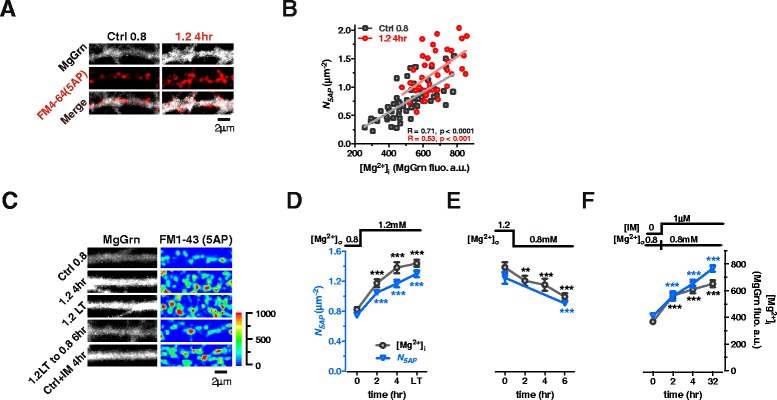
Functional terminal density to bursts is regulated by intracellular Mg^2+^ level at individual branches. (**a**) Colocalization of MgGrn-marked [Mg^2+^]_i_ and FM4-64-marked functional terminals responding to 5AP bursts input at single branches before (Ctrl 0.8) and after elevating [Mg^2+^]_o_ for 4 hr (1.2 4hr). (**b**) *N*_*5AP*_ was linearly correlated with [Mg^2+^]_i_ (normalized fluorescent intensity of MgGrn) at individual branches before and after elevating [Mg^2+^]_o_ for 4 hr. Each point represents the data from a branch. (**c**) [Mg^2+^]_o_ ‘ON’ and ‘OFF’ experiment. MgGrn marked [Mg^2+^]_i_ and FM1-43 marked functional terminals responding to 5AP input. “1.2 LT”: elevating [Mg^2+^]_o_ from 0.8 to 1.2 mM for > 48 hr; “1.2 LT to 0.8 6hr”: decreasing [Mg^2+^]_o_ from 1.2 to 0.8 mM for 6 hr; “Ctrl + IM 4hr”: adding 1 μM Imipramine (IM) into Ctrl ([Mg^2+^]_o_ 0.8 mM) for 4 hr. Pseudo-color scale: fluorescent intensity. (**d**-**f**) The time-course curves of [Mg^2+^]_i_ and *N*_*5AP*_ by different treatments shown (**c**) (n = 5 coverslips for each point). For (**c**-**f**), data from sister cultures of the same batch. For (**d**-**f**), the mean ± SEM of coverslips was presented. Two-tailed Student’s t-test comparing each time point after [Mg^2+^]_o_ change to initial [Mg^2+^]_o_, *** p < 0.001

To check this hypothesis, we chose [Mg^2+^]_o_ of 0.8 mM as the baseline concentration (Ctrl 0.8) and elevated [Mg^2+^]_o_ from 0.8 to 1.2 mM for 4 hr (1.2 4hr). We then marked intracellular Mg^2+^ with MgGrn and functional terminals (responding to 5AP bursts) with FM4-64 and colocalized FM4-64(+) puncta and MgGrn(+) fluorescence at each branch (Fig. 2a, left panel). Quantitative analysis revealed that *N*_*5AP*_ was linearly correlated with [Mg^2+^]_i_ at individual branches (Fig. 2b black squares, linear regression) at control conditions. After elevating [Mg^2+^]_o_ for 4 hr, both the density of FM4-64(+) puncta and MgGrn(+) fluorescence increased (Fig. 2a, right panel), and remained linearly correlated (Fig. 2b red circles, linear regression). This result demonstrated that the functional terminal density is closely matched with [Mg^2+^]_i_ at different branches in the same network. Because of this positive correlation, we hypothesized that [Mg^2+^]_i_ might play a pivotal role in the regulation of *N*_*5AP*_.

To verify the hypothesis, we performed two types of experiments using sister cultures from the same experimental batch of cultured neurons. First, we examined the temporal correlation of [Mg^2+^]_i_ and *N*_*5AP*_ by performing a [Mg^2+^]_o_ ‘ON’ and ‘OFF’ experiment and comparing the time-course curves of [Mg^2+^]_i_ and *N*_*5AP*_. We found that the increase in *N*_*5AP*_ matched temporally with the increase in [Mg^2+^]_i_ after elevating [Mg^2+^]_o_ from 0.8 to 1.2 mM. Maximum *N*_*5AP*_ was reached after 4 hr as the number of *N*_*5AP*_ at 4 hr persisted permanently (48 hr to 2 weeks) (Fig. 2c and d). In contrast, *N*_*5AP*_ decreased following the reduction of [Mg^2+^]_o_ from 1.2 to 0.8 mM for 6 hr (Fig. 2c and e). Second, we examined the effects of [Mg^2+^]_i_ elevation via a chemical agent - Imipramine (IM), to rule out the possibility that increasing [Mg^2+^]_o_ could increase Ca^2+^ channel blockage, thereby contributing to the increase in functional terminal density. IM, which can increase [Mg^2+^]_i_ by blocking efflux of Mg^2+^ through Mg^2+^ channels [53], was administered at 1 μM for 4 hr. Following 4 hr IM administration, *N*_*5AP*_ was increased in concert with the elevation of [Mg^2+^]_i_, and the enhancement persisted for ~32 hr (Fig. 2c and f). Based on these data, we concluded that [Mg^2+^]_i_ might be an important factor in the regulation of functional terminal density.

### Low presynaptic Ca^2+^ sensitivity can account for the nonfunctional terminals

By means of manipulation of [Mg^2+^]_i_ by [Mg^2+^]_o_, *N*_*5AP*_ could be conveniently and reversibly altered under physiological conditions. Since 4 hr [Mg^2+^]_o_ elevation was effective at increasing *N*_*5AP*_ (Fig. 2d), we used [Mg^2+^]_o_ elevation in neuronal cultures as a tool to determine the molecular substrates involved in *N*_*5AP*_ regulation.

**Fig. 3:**
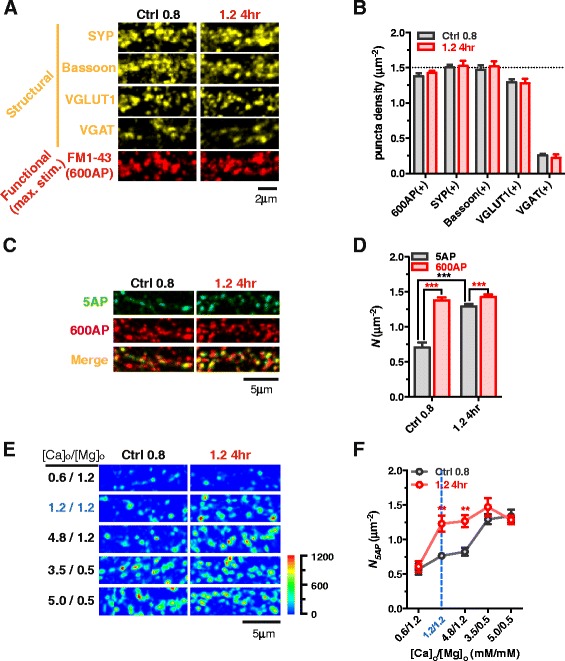
Presynaptic Ca^2+^ sensitivity determines functional terminal density to bursts. (**a** and **b**) At low (Ctrl 0.8) and high [Mg^2+^]_o_ (1.2 4hr) conditions, colocalization of SYP, VGLUT1, VGAT and Bassoon positive fluorescent puncta with FM1-43 labeled releasable terminals to “maximal stimulation” (600 APs at 10 Hz) at the same dendrite (**a**). No significant changes were observed (n = 6 coverslips for each bar, p = 0.38-0.84) (**b**). Dotted line represents the total number of SYP(+) puncta of Ctrl 0.8. (**c** and **d**) Colocalization of FM(+) terminals following 5AP- and 600AP-stimulation at the same branches (**c**). 51.3 ± 9.8 % (n = 12 coverslips, totally 7542 5AP-induced puncta and 14701 600AP-induced puncta were analyzed) and 90.6 ± 2.9 % (n = 16 coverslips, totally 16926 5AP-induced puncta and 18601 600AP-induced puncta were analyzed) terminals were functional in response to 5AP bursts (**d**) at low and high [Mg^2+^]_o_ conditions. (**e** and **f**) Acute change of [Ca^2+^]_o_/[Mg^2+^]_o_ ratio led to the change of detectable functional terminals (**e**). Statistics of *N*_*5AP*_ at different [Ca^2+^]_o_/[Mg^2+^]_o_ ratio (**f**) (n = 5–11 coverslips). Pseudo-color scale: fluorescent intensity. Two-tailed Student’s t-test comparing 5AP to 600AP as indicated (**d**), or comparing 1.2 4hr to Ctrl 0.8 at each [Ca^2+^]_o_/[Mg^2+^]_o_ (**f**), ** p < 0.01, *** p < 0.001. For each bar or point in (**b**, **d** and **f**), the mean ± SEM of coverslips was presented

To start, we examined whether the increase in *N*_*5AP*_ was caused by synaptogenesis. By immunofluorescence (IF), we labeled structural terminals with antibodies against several presynaptic proteins, including synaptophysin (SYP, vesicle protein), VGLUT1 (excitatory vesicle protein), VGAT (inhibitory vesicle protein) and Bassoon (active zone protein) (Fig. 3a). There were no significant changes in the density of presynaptic protein puncta after elevating [Mg^2+^]_o_ for 4 hr (Fig. 3b), suggesting that elevation of [Mg^2+^]_i_ might not, at least within 4 hr, induce synaptogenesis.

Next, we evaluated the functionality of these terminals. We applied a “maximal stimulation”, 600 APs at 10 Hz (600AP) [54], to determine the vesicle turnover ability of terminals. At the low [Mg^2+^]_o_ condition, almost all available structural terminals had the ability to release vesicles under maximal stimulation (Fig. 3a). The density of FM(+) puncta (1.38 μm^−2^) was close to that of structure-protein positive puncta (SYP(+) 1.50 and Bassoon(+) 1.47 μm^−2^), indicating almost all terminals are functional under maximal stimulation (Fig. 3b).

Notably, at the low [Mg^2+^]_i_ condition, the 5AP bursting input-induced FM(+) puncta density was remarkably lower (0.73 μm^−2^, Fig. 2d control) than the 600AP-induced FM(+) puncta density (1.38 μm^−2^). It is possible that this difference was due to the ability of terminals to release vesicles under maximal stimulation but not under 5AP bursting input. To test this possibility, we compared the vesicle release of terminals under 5AP and 600AP stimulations at the same dendrite (Fig. 3c). Indeed, only ~50 % of terminals were activated under 5AP stimulation at the low [Mg^2+^]_o_ condition (Fig. 3c and d, Ctrl 0.8), while ~90 % of terminals were functional under 5AP stimulation after elevating [Mg^2+^]_o_ for 4 hr (Fig. 3c and d, 1.2 4hr).

We then determined what cellular processes were modified after elevation of [Mg^2+^]_i_. One of the prominent differences between 5AP and 600AP stimulation is the resultant amount of presynaptic Ca^2+^ influx. At the low [Mg^2+^]_o_ condition, if terminals have low Ca^2+^ sensitivity, they might not be able to release vesicles in response to 5AP-induced Ca^2+^ influx, but able to release vesicles under the much higher 600AP-induced Ca^2+^ influx. If this hypothesis was true, elevation of [Ca^2+^]_o_/[Mg^2+^]_o_, which is a classic approach to enhance presynaptic Ca^2+^ influx [55], should be able to turn nonfunctional terminals into functional ones under 5AP bursting stimulation. Experimentally, we acutely manipulated the [Ca^2+^]_o_/[Mg^2+^]_o_ in working solution right before FM dye staining. At the low [Mg^2+^]_o_ condition (Ctrl 0.8), raising the [Ca^2+^]_o_/[Mg^2+^]_o_ from 1 (normal working solution, [Ca^2+^]_o_ 1.2 and [Mg^2+^]_o_ 1.2 mM) (Fig. 3e and f, blue ink and dotted line) to 4 ([Ca^2+^]_o_ 4.8 and [Mg^2+^]_o_ 1.2 mM) induced no increase in *N*_*5AP*_ (Fig. 3e and f, Ctrl 0.8). However, strikingly, when [Ca^2+^]_o_/[Mg^2+^]_o_ was ≥ 7, *N*_*5AP*_ increased dramatically up to levels comparable to those at the high [Mg^2+^]_o_ condition (1.2 4hr) (Fig. 3e and f). These data suggest that the primary effect of elevating [Mg^2+^]_i_ on the functionality of terminals might be the enhancement of presynaptic Ca^2+^ sensitivity. If so, a decrease in Ca^2+^ influx in terminals at the high [Mg^2+^]_o_ condition (1.2 4hr) might lead to the neutralization of the increase in *N*_*5AP*_. Indeed, reducing [Ca^2+^]_o_/[Mg^2+^]_o_ to 0.5 ([Ca^2+^]_o_ 0.6 and [Mg^2+^]_o_ 1.2 mM) led to a significant decrease in *N*_*5AP*_ in comparison with that under normal [Ca^2+^]_o_/[Mg^2+^]_o_ (Fig. 3e and f), showing that Mg^2+^-induced enhancement of functional terminal density is Ca^2+^ influx-dependent. Note that when [Ca^2+^]_o_/[Mg^2+^]_o_ was at extreme (0.5 or ≥ 7), there were no differences in *N*_*5AP*_ at the low (Ctrl 0.8) and high [Mg^2+^]_o_ conditions (1.2 4hr).

Taking these results together, we concluded that (1) at low [Mg^2+^] conditions (e.g. Ctrl 0.8), approximately half of the terminals failed to release vesicles in response to physiological patterns of input and are thereby in a nonfunctional state; (2) the non-function is due to low presynaptic Ca^2+^ sensitivity, which can be ameliorated by increasing Ca^2+^ influx by either boosting temporal intensity of stimuli or elevating [Ca^2+^]_o_/[Mg^2+^]_o_ ratio; (3) elevation of [Mg^2+^]_i_ can enhance presynaptic Ca^2+^ sensitivity such that most terminals are capable of releasing vesicles to physiological stimuli, leading to higher functional terminal density at dendritic branches.

### The quantity of Ca^2+^-sensitivity-related proteins in presynaptic terminals determines the terminal functionality under physiological conditions

**Fig. 4:**
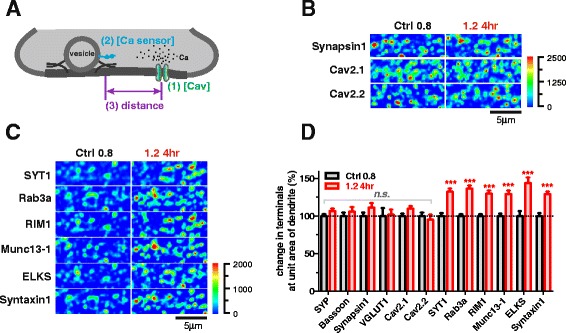
Elevating [Mg^2+^]_i_ leads to increased Ca^2+^-sensitivity-related proteins in terminals. (**a**) Schematic cartoon illustrates the three critical biophysical factors affecting presynaptic vesicle release. (**b** and **c**) Immunofluorescence. No change of quantity of Synapsin1, Ca_v_2.1 and Ca_v_2.1 (**b**), but remarkable increase in Ca^2+^-sensitivity-related proteins at dendritic branches (**c**) after elevating [Mg^2+^]_o_ for 4 hr. Pseudo-color scale: fluorescent intensity. (**d**) Elevating [Mg^2+^]_o_ for 4 hr led to increase in Ca^2+^-sensitivity-related protein positive fluorescence in terminals at dendritic branches (n = 10–21 coverslips for each point). The mean ± SEM of coverslips was presented. Two-tailed Student’s t-test, comparing 1.2 4hr to Ctrl 0.8, *n.s.* no significance, *** p < 0.001

To further understand how [Mg^2+^]_i_ affects presynaptic Ca^2+^ sensitivity, we investigated the potential molecular mechanisms underlying this phenomenon. Generally, Ca^2+^ sensitivity of presynaptic terminals is determined by three biophysical factors (illustrated in Fig. 4a): (1) the quantity of presynaptic Ca^2+^ channels [56] (mainly Ca_v_2.1 and Ca_v_2.2 in hippocampal terminals [57]), which conduct the Ca^2+^ influx; (2) the quantity of Ca^2+^ sensor proteins (mainly Synaptotagmin1 [SYT1] for excitatory central synapses [58]), which couple the Ca^2+^ signals to vesicle release; and (3) the distance from Ca^2+^ channels to vesicle release machinery, which determines the coupling efficacy of the Ca^2+^ influx to the operation of release machinery. The coupling efficacy is regulated by multiple vesicle and active zone proteins (e.g. Rab3, RIM1, Munc13, ELKS, Syntaxin1) [44].

To address these three possibilities, we compared the profiles of protein expression in terminals at the low (Ctrl 0.8) versus the high [Mg^2+^]_o_ condition (1.2 4hr) using IF staining. There was no difference in the expression level of Ca_v_2.1 and Ca_v_2.2 per area of dendrites (Fig. 4b and d), eliminating the first possibility. However, the expression of Ca^2+^ sensor protein (SYT1) and coupling proteins (e.g. Rab3a, RIM1, Munc13-1, ELKS, Syntaxin1) were significantly higher in terminals at the high [Mg^2+^]_o_ condition (30-44 %, p < 0.001) (Fig. 4c and d). These data, together with the observation that the expression of structural (vesicle and active zone) presynaptic proteins (SYP, Bassoon, VGLUT1 and Synapsin1) did not change (Figs. 3a and b, 4b) suggest that elevation of [Mg^2+^]_i_ increased presynaptic Ca^2+^ sensitivity via enhancing the expression of presynaptic proteins critical for controlling presynaptic Ca^2+^ efficacy (we refer to these proteins hereafter as presynaptic Ca^2+^-sensitivity-related proteins).

### Role of [Mg^2+^]_i_ in controlling the efficiency of axonal transport of Ca^2+^-sensitivity-related proteins within several hours

We carried out further experiments to explore how elevating [Mg^2+^]_i_ promoted the augmentation of Ca^2+^-sensitivity-related proteins in terminals. Generally, the quantity of proteins in terminals depends on the three major processes involved in the protein life cycle: (1) synthesis, (2) transport, and (3) degradation, so we investigated if [Mg^2+^]_i_ affected any of these processes in regard to Ca^2+^-sensitivity-related proteins in terminals.

**Fig. 5:**
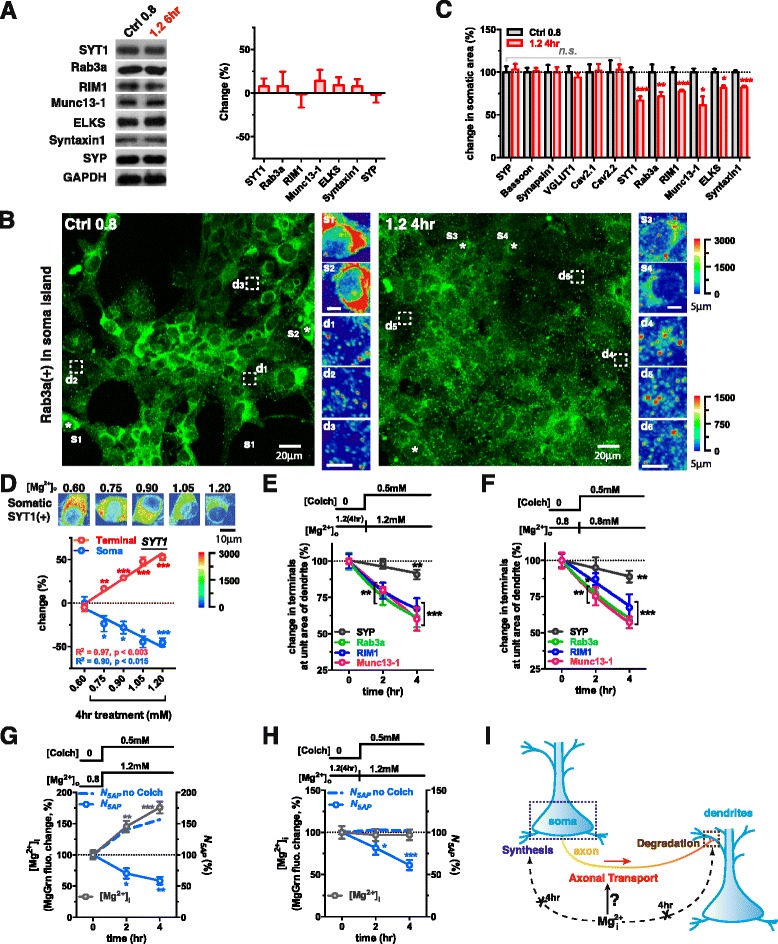
Elevating [Mg^2+^]_i_ might increase the efficiency of axonal transport of Ca^2+^-sensitivity-related proteins. (**a**) Western blot detection showed no significant increase in the total protein levels of each Ca^2+^-sensitivity-related protein or SYP after elevating [Mg^2+^]_o_ for 6 hr (n = 12 coverslips from 4 batches). (**b**) The immunoreactivity of Rab3a in a soma island from the culture coverslips before and after elevating [Mg^2+^]_o_ for 4 hr. The pseudo-colored images were the magnifications of the local somatic or terminal regions marked in the large images. Stars s_1_-s_4_: examples of immunoreactive cell bodies. Dashed boxes d_1_-d_6_: representative terminal regions. Pseudo-color scale: fluorescent intensity; the upper one was for s_1_-s_4_ and the lower one was for d_1_-d_6_. (**c**) Significant decreases in Ca^2+^-sensitivity-related proteins in somatic area after elevating [Mg^2+^]_o_ for 4 hr (analyzed from the same raw data as in Fig. 4d, 50–98 cell bodies). Dotted line represents levels of proteins at [Mg^2+^]_o_ 0.8 normalized to 100 %. (**d**) Quantity of immunostained SYT1 at terminals (n = 6–8 coverslips for each data bar) or soma (40–55 cells from the same AOIs) changed in opposite directions after elevating [Mg^2+^]_o_ from 0.6 to 0.75-1.2 mM (in gradient) for 4 hr. Dotted line represents initial SYT1 at Terminal and Soma, normalized to 0 % change. Pseudo-colored images represented the immunoreactivity in somatic area at different [Mg^2+^]_o_ levels. Pseudo-color scale: fluorescent intensity. (**e** and **f**) Blocking axonal transport by 0.5 mM Colchicine (Colch) caused a decrease in proteins in terminals at low (**e**) and high [Mg^2+^]_o_ conditions (**f**) (n = 8–10 coverslips for **e** and 10–15 coverslips for **f**). Dotted lines represent initial protein levels before addition of Colch, normalized to 100 %. (**g** and **h**) Blocking axonal transport by 0.5 mM Colch led to a decrease in *N*_*5AP*_ but no effect on [Mg^2+^]_i_ compared to not adding Colch (blue dashed lines) (n = 5–6 coverslips for **g** and 5–8 coverslips for **h**). Dotted line represented the initial [Mg^2+^]_i_ and *N*_*5AP*_ normalized to 100 %. (**i**) Schematic cartoon: intracellular Mg^2+^ might majorly affect axonal transport efficiency, but not affect protein synthesis or degradation within a few hours. The mean ± SEM of all coverslips was presented. Two-tailed Student t-test for comparing 1.2 4hr to Ctrl 0.8 (**c**) and comparing pre and post treatment values (**d**-**h**), * p < 0.05, ** p < 0.01, *** p < 0.001

First, we compared the quantity of SYP and Ca^2+^-sensitivity-related proteins from total proteins extracted from entire neurons at the low (Ctrl 0.8) and high [Mg^2+^]_o_ (1.2 6 hr) conditions by Western blot. Surprisingly, the quantity of each protein at the high [Mg^2+^]_o_ condition was not significantly higher (at least within 6 hr) than that at the low [Mg^2+^]_o_ condition (Fig. 5a), ruling out the possibility that elevating [Mg^2+^]_i_ could promote the detectable enhancement of synthesis of Ca^2+^-sensitivity-related proteins rapidly (within 6 hr).

Next, we checked whether elevating [Mg^2+^]_i_ could promote the transport of Ca^2+^-sensitivity-related proteins from soma to axonal terminals. Interestingly immunoreactive fluorescence for the Ca^2+^-sensitivity-related protein Rab3(+) decreased in somatic area (Fig. 5b s_1_-s_2_ vs. s_3_-s_4_) but increased in terminals (Fig. 5b d_1_-d_3_ vs. d_4_-d_6_). This result was also found for other Ca^2+^-sensitivity-related proteins tested (Fig. 5c; from the same experiment as in Fig. 4d). Conversely, after 4 hr high [Mg^2+^]_o_, those proteins whose immunoreactivity changed little in terminals at dendrites (SYP, Bassoon, Synapsin1, VGLUT1, Ca_v_2.1 and Ca_v_2.2) (Fig. 4d) also had constant immunoreactivity in the somatic area (Fig. 5c, from the same experiments as in Fig. 4d). These data highly suggested that elevation of [Mg^2+^]_i_ might accelerate the transport of Ca^2+^-sensitivity-related proteins from soma to terminals selectively. To study this phenomenon quantitatively, we examined the changes of the quantity of SYT1 in the somatic area and terminals at dendrites by IF after elevating [Mg^2+^]_o_ from 0.6 to 0.75-1.2 mM in gradient for 4 hr (thereby [Mg^2+^]_i_ could be clamped at different levels). The immunoreactivity of SYT1 in the somatic area (Fig. 5d, upper) and terminals at dendrites changed in opposite directions, linearly proportional to the Mg^2+^ level (Fig. 5d, linear regression).

Third, we examined the effects of elevating [Mg^2+^]_i_ on the rate of protein degradation in terminals. Assuming that presynaptic terminals do not have the capacity to synthesize new Ca^2+^-sensitivity-related proteins (no evidence has been found that they do), the quantity of presynaptic Ca^2+^-sensitivity-related proteins at equilibrium would be largely determined by the balance of protein transport and degradation. Therefore, if we blocked axonal transport, the rate of protein decline would reflect the rate of protein degradation. When axonal transport was blocked by 0.5 mM Colchicine (Colch) [59], the concentration of the presynaptic proteins studied decreased linearly, as a function of the amount of blocking time (Fig. 5e and f), albeit at varying rates. The rate of protein degradation (half-life) for the Ca^2+^-sensitivity-related proteins (e.g. Rab3a, RIM1 and Munc13-1) (several hours) was much faster than that for structure-protein SYP (several days). Interestingly, the elevation of [Mg^2+^]_i_ did not alter the degradation rate of presynaptic proteins, indicating that degradation rate was not part of the mechanism by which increased [Mg^2+^]_i_ altered presynaptic Ca^2+^ sensitivity (Fig. 5e and f).

Altogether, these data suggested that the Ca^2+^-sensitivity-related proteins had a relatively fast degradation rate and thereby their quantities in terminals at dendrites were strongly dependent upon the efficiency of axonal transport. The elevation of [Mg^2+^]_i_ likely promoted the augmentation of Ca^2+^-sensitivity-related proteins in terminals by increasing the efficiency of their transport from soma to terminals, which ensured sufficient Ca^2+^ sensitivity for AP-dependent vesicle turnover (illustrated in Fig. 5i).

Based on these data, we inferred that impairment of axonal transport would reduce the density of functional terminals immediately (within hours). To validate this, we measured the temporal changes of *N*_*5AP*_ after blockade of axonal transport by Colch. Colch treatment did not prevent elevation of [Mg^2+^]_i_ after increasing [Mg^2+^]_o_ but it prevented the increase in *N*_*5AP*_ (Fig. 5g). Furthermore, blocking axonal transport at the high [Mg^2+^]_o_ condition caused a remarkable reduction of *N*_*5AP*_, without change of [Mg^2+^]_i_ (Fig. 5h). These data demonstrate the critical role of ongoing axonal transport in maintaining functional status of terminals.

### Local energy supply is enhanced by elevation of intracellular Mg^2+^

The above studies suggest that the level of intracellular Mg^2+^ may determine the efficiency of protein transport in axons. Previous studies indicate that [Mg^2+^]_i_ and intracellular ATP concentration ([ATP]_i_) are two endogenous factors critical for controlling the speed of axonal transport [60]. Therefore, the elevation of [Mg^2+^]_i_ might improve the efficiency of axonal transport by affecting the efficacy of motor proteins [60], and/or indirectly by promoting energy supply, as Mg^2+^ is well known to be necessary for normal mitochondrial function.

**Fig. 6:**
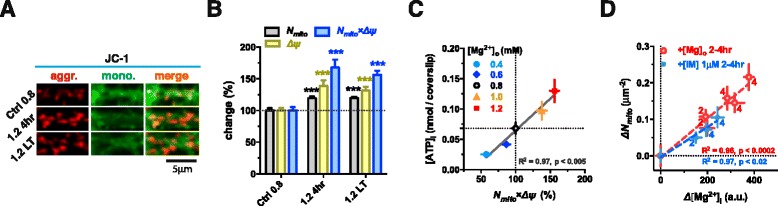
Elevating [Mg^2+^]_i_ increases general mitochondrial function and intracellular ATP. (**a**) The mitochondrial potential (*ΔΨ*) was determined by the ratio of aggregate and monomer of JC-1 fluorescence. (**b**) Both mitochondrial density (*N*_*mito*_) and general mitochondrial function (*N*_*mito*_ × *ΔΨ*) in distal branches were significantly enhanced after elevating Mg^2+^ level for 4 hr (19.4 ± 2.9 % and 67.8 ± 12.4 %, n = 14 coverslips) and for long term (>48 hr) (19.6 ± 2.1 % and 56.2 ± 6.5 %, n = 20 coverslips). Dotted line represents initial value of *N*_*mito*_, *N*_*mito*_ × *ΔΨ* and *ΔΨ* normalized to 100 %. (**c**) Intracellular ATP concentration ([ATP]_i_) was linearly correlated with *N*_*mito*_ × *ΔΨ* at equilibrium (altering [Mg^2+^]_o_ for > 12 hr) (n = 7 coverslips). (**d**) Absolute temporal changes of *N*_*mito*_ (*ΔN*_*mito*_) and [Mg^2+^]_i_ (Δ[Mg^2+^]_i_) exhibited a linear correlation, either by elevating [Mg^2+^]_o_ or by administering 1 μM Imipramine (IM) for 2–4 hr (n = 5–11 coverslips). Each point represented the changes of both *ΔN*_*mito*_ and Δ[Mg^2+^]_i_ (using sister cultures from the same batch) at different time after a treatment, and the colored numbers beside each point indicated the hours after the treatment. These data were collected from 2 individual batches of measurements. The mean ± SEM of coverslips was presented. Two-tailed Student t-test for (**b**) comparing both 1.2 4hr and 1.2 LT to Ctrl 0.8, *** p < 0.001. Linear regression for (**c** and **d**)

We studied the effects of elevating [Mg^2+^]_i_ on the spatial distribution and function of mitochondria. The function of each mitochondrion was quantified by measuring its membrane potential (*ΔΨ*), using the *ΔΨ*-sensitive fluorescent dye JC-1 [61]. *ΔΨ* increased by ~39 % after elevating Mg^2+^ for 4 hr and this increase persisted up to several weeks (Fig. 6a and b). The number of mitochondria per unit area of distal dendritic branches (*N*_*mito*_) also increased, by ~20 % (Fig. 6b). Since *N*_*mito*_ and *ΔΨ* are two key parameters for the determination of mitochondrial function, we used the product of *N*_*mito*_ and *ΔΨ* to represent the total mitochondrial function per unit area of dendritic branches in the synaptic network, and found *N*_*mito*_ × *ΔΨ* increased by ~68 % after elevating [Mg^2+^]_o_ for 4 hr (Fig. 6b).

Next, we examined if increased *N*_*mito*_ × *ΔΨ,* after elevation of [Mg^2+^]_o_ led to increased [ATP]_i_. We set the range of [Mg^2+^]_o_ at 0.4-1.2 mM for more than 12 hr before measuring [ATP]_i_ to ensure equilibrium of the cytoplasmic ATP concentration. Indeed, as *N*_*mito*_ × *ΔΨ* increased linearly with the elevation of [Mg^2+^]_o_, [ATP]_i_ also increased linearly with *N*_*mito*_ × *ΔΨ* (Fig. 6c). To directly show that the extent of [Mg^2+^]_i_ influenced mitochondrial function at the local area of distal branches, we plotted the correlation between the change of [Mg^2+^]_i_ (Δ[Mg^2+^]_i_) and the change of *N*_*mito*_ (*ΔN*_*mito*_*)*. Indeed, *ΔN*_*mito*_ was linearly correlated with Δ[Mg^2+^]_i_ (Fig. 6d).

These data suggest that the elevation of [Mg^2+^]_i_ increased energy supply in local area of the network.

### Local energy supply, Ca^2+^-sensitivity-related proteins and functional terminal density

**Fig. 7:**
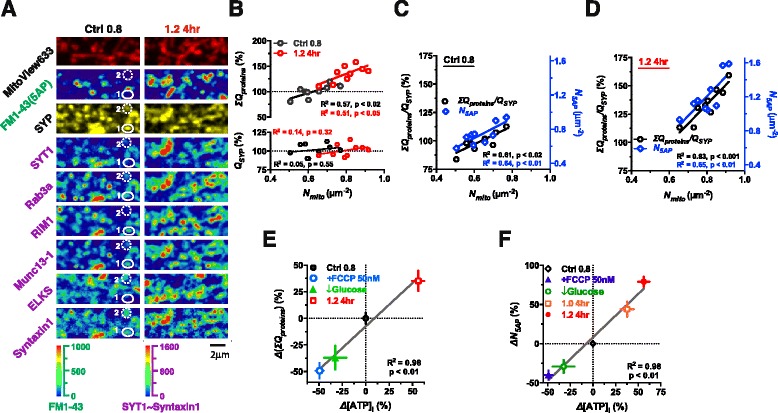
Linear correlations between local energy supply, Ca^2+^-sensitivity-related proteins in terminals and functional terminal density. (**a-d**) Co-staining and quantitative analysis of mitochondria, functional terminals and presynaptic proteins. (**a**) At the low and high [Mg^2+^]_o_ conditions, mitochondria, functional terminals and Ca^2+^-sensitivity-related proteins were marked at the same dendritic branch. The numbers 1 and 2 indicated the positions of two nonfunctional terminals marked by white circles. (**b**) No correlation between normalized quantity (*Q*) of SYP (*Q*_*SYP*_) and *N*_*mito*_, whereas linear correlation between total amount of Ca^2+^-sensitivity-related proteins (*ΣQ*_*proteins*_) and *N*_*mito*_. (**c** and **d**) Relative quantity of Ca^2+^-sensitivity-related proteins to structure-related protein SYP, i.e. *ΣQ*_*proteins*_ /*Q*_*SYP*_, was linearly correlated with *N*_*mito*_ (**c** and **d**, black circles), meanwhile the density of functional terminals (*N*_*5AP*_) was also linearly correlated with *N*_*mito*_ (blue diamonds) at the low (**c**) and high [Mg^2+^]_o_ conditions (**d**) (n = 9 AOIs from 1 coverslip for each group, the two coverslips were from the same culturing dish). Each point represented an AOI for (**b-d**). Pseudo-color scale: fluorescent intensity. (**e**) The change of *ΣQ*_*proteins*_ (Δ*ΣQ*_*proteins*_) was linearly correlated with the change of [ATP]_i_ (Δ[ATP]_i_) after different treatments. (**f**) Δ*N*_*5AP*_ and Δ[ATP]_i_ exhibited linear correlation after different treatments. For (**e** and **f**), the treatments included adding 50 nM FCCP for 16 hr, reducing Glucose concentration from 28 to 2 mM in culture medium for 12 hr and elevating [Mg^2+^]_o_ from 0.8 to 1.0 or 1.2 mM for 4 hr (n = 4–6 coverslips for **e**; n = 5–8 coverslips for **f**). The percentage in (**e** and **f**) was normalized to the mean of Ctrl 0.8 group (0 %). The experiments were performed using sister cultures for each treatment. The mean ± SEM of coverslips was presented. Linear regression for (**b**-**f**)

We investigated whether the increased quantity of Ca^2+^-sensitivity-related proteins at terminals and subsequently the functionality of terminals as a result of elevated [Mg^2+^]_i_ was due to enhanced mitochondrial function and local energy supply at dendritic branches. To address this, we examined whether the quantity of Ca^2+^-sensitivity-related proteins or functional terminal density was correlated with mitochondrial density since both are upregulated after the elevation of [Mg^2+^]_i_ (Figs. 4d, 6b and d). Mitochondria were stained with the fluorescent marker MitoView633. Then, at the same region, functional terminals responding to 5AP bursts were labeled by FM1-43. Subsequently, structure- and Ca^2+^-sensitivity-related proteins in terminals were stained by IF at the low and high [Mg^2+^]_o_ conditions (Fig. 7a). We observed that the nonfunctional terminals (the SYP positive but FM1-43 negative puncta) usually lacked some of the Ca^2+^-sensitivity-related proteins, but the type of proteins whose expression was low varied in different nonfunctional terminals. For example, in terminal 1 of Fig. 7a, the expression of SYT1 and ELKS was relatively low, while in terminal 2, the expression of Rab3a, RIM1 and Munc13-1 was relatively low (Fig. 7a white circles: terminal 1, 2). This observation suggested that it was the inadequate expression of multiple Ca^2+^-sensitivity-related proteins, but not specific one of them that made a presynaptic terminal nonfunctional. Therefore, we used *ΣQ*_*proteins*_ to represent the total quantity of Ca^2+^-sensitivity-related proteins (see Methods).

When we compared the relationship of *N*_*mito*_ with the quantity of the structure-related protein SYP (*Q*_*SYP*_) and with *ΣQ*_*proteins*_, we found that *ΣQ*_*proteins*_ was correlated with *N*_*mito*_, while *Q*_*SYP*_ was not (Fig. 7b lower). Elevating [Mg^2+^]_o_ increased both *ΣQ*_*proteins*_ and *N*_*mito*_ (Fig. 7b-d). The correlation between *ΣQ*_*proteins*_, the ratio of *ΣQ*_*proteins*_*/Q*_*SYP*_ and *N*_*mito*_ remained (Fig. 7b upper, c and d). Most importantly, *N*_*5AP*_ at the same local area was also linearly correlated with *N*_*mito*_ (Fig. 7c and d). Therefore, the close linear correlations between *ΣQ*_*proteins*_, *ΣQ*_*proteins*_/*Q*_*SYP*_, *N*_*5AP*_ and *N*_*mito*_ support the notion that local energy supply provided by mitochondria might be important for maintaining high levels of Ca^2+^-sensitivity-related proteins in terminals, which in turn determine the density of functional terminals.

To demonstrate the causal relationship between energy supply and *N*_*5AP*_, we tested whether modification of energy supply would affect both *ΣQ*_*proteins*_ and *N*_*5AP*_ concurrently. Energy supply was increased by elevating [Mg^2+^]_o_ (from 0.8 to 1.0 or 1.2 mM for 4 hr), or decreased by either adding FCCP (disturbs mitochondrial function by preventing the H^+^-coupling of respiratory chain) or by lowering the extracellular glucose concentration (from 28 to 2 mM) for 12 hr in sister cultures from the same batch of cultured neurons, and then [ATP]_i_, *ΣQ*_*proteins*_ and *N*_*5AP*_ under these conditions were compared. Using cultures under the low [Mg^2+^]_o_ condition (Ctrl 0.8) as a control, we plotted the relationship between the change of [ATP]_i_ and the change of *ΣQ*_*proteins*_ and *N*_*5AP*_. The increase or decrease in [ATP]_i_ was linearly correlated with the increase or decrease in *ΣQ*_*proteins*_ and *N*_*5AP*_ (Fig. 7e and f).

Taking all the data together, we conclude that local energy supply is one of major factors that determine functional terminal density.

### Correlation between quantity of Ca^2+^-sensitivity-related proteins and mitochondrial density in intact animals

Finally, we determined whether the *in vitro* findings could be verified in intact animals. We treated mature male *Sprague–Dawley* rats (16 months old) with Magnesium L-Threonate (MgT) in drinking water. This treatment is known to be effective in elevating [Mg^2+^]_CSF_ [62]. After 8 months of MgT supplement (24 months old), we sacrificed the animals, dissected the Hippocampus CA1 Stratum Radiatum region of the brain, and conducted both electron microscopy (EM) and IF.

**Fig. 8:**
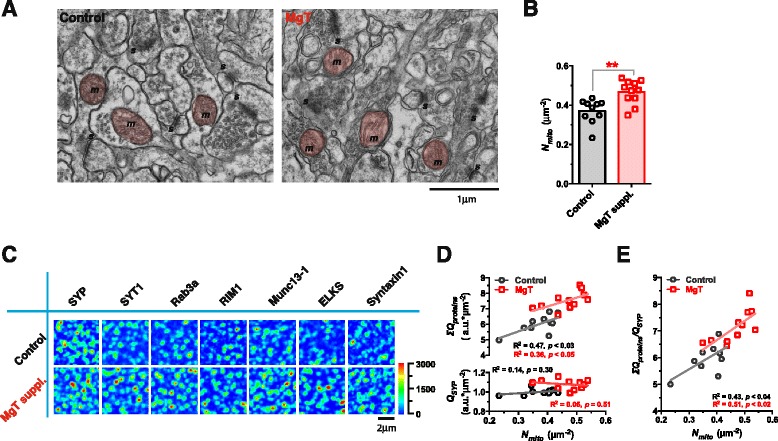
Quantity of Ca^2+^-sensitivity-related proteins versus density of mitochondria in intact animals. (**a**) Electron Microscopy (EM) images of Hippocampus CA1 Stratum Radiatum region (HP/CA1/SR) from 24-months-old Control and MgT-supplemented (for 8 months) rats (*m*, mitochondrion [red colored]; *s*, synapse). (**b**) Density of mitochondria (*N*_*mito*_, number of mitochondria per area of 70 nm brain slice) in MgT group (0.47 ± 0.02 μm^−2^, n = 11 rats) was 25.9 % higher than Control group (0.37 ± 0.02 μm^−2^, n = 9 rats) (Kolmogorov-Smirnov test, p < 0.01). (**c**) Immunostained structure-related protein SYP and Ca^2+^-sensitivity-related proteins in 70 nm ultrathin slices from adjacent tissue blocks in HP/CA1/SR of the same rats as in (**a **and **b**). Quantity of Ca^2+^-sensitivity-related proteins was higher in MgT group than in Control group. Pseudo-color scale: fluorescent intensity. (**d**) Normalized total quantity of each protein (*Q*) was calculated for each rat. *Q*_*SYP*_ was not correlated with *N*_*mito*_ (**d** lower) Total amount of Ca^2+^-sensitivity-related proteins (*ΣQ*_*proteins*_) was linearly correlated with *N*_*mito*_ for both groups and the correlation curve was positively shifted in MgT group (**d** upper) (Kolmogorov-Smirnov test, p < 0.0005). (**e**) Relative quantity of Ca^2+^-sensitivity-related proteins to SYP, i.e. *ΣQ*_*proteins*_/*Q*_*SYP*_, was linearly correlated with *N*_*mito*_, and the linear correlation was positively shifted in MgT group (Kolmogorov-Smirnov test, p < 0.003). Linear regression for (**d **and **e**)

First we checked whether MgT supplementation could modify mitochondrial density (*N*_*mito*_). We measured *N*_*mito*_ (number of mitochondria per unit area of image) in EM images of each animal (Fig. 8a). Mean *N*_*mito*_ in MgT group (0.47 ± 0.02 μm^−2^, N = 11 rats) was ~26 % higher than that in control group (0.37 ± 0.02 μm^−2^, N =10 rats) (Kolmogorov-Smirnov test, p < 0.01) (Fig. 8b). The extent of the increase in *N*_*mito*_ by MgT supplementation in intact animals was similar to that in cultured neurons (Fig. 7b).

Next, we checked the expression of structure-related (e.g. SYP) and Ca^2+^-sensitivity-related proteins (e.g. SYT1, Rab3a, RIM1, Munc13-1, ELKS and Syntaxin1) in 70 nm ultrathin slices by IF. The total fluorescent intensity of immunoreactive puncta of Ca^2+^-sensitivity-related proteins was higher in MgT group than that in control group (Fig. 8c). We calculated the quantity of each protein (*Q*) in each individual rat, and found no correlation between *Q*_*SYP*_ and *N*_*mito*_ for each rat in both groups (Fig. 8d lower). However, *ΣQ*_*proteins*_ (reactive Ca^2+^-sensitivity-related proteins) was linearly correlated with *N*_*mito*_ in both groups, and MgT treatment positively shifted the correlation curve (Fig. 8d upper). Thus, the data from intact rats were in agreement with the *in vitro* data (Fig. 7b). On average, *ΣQ*_*proteins*_ was ~25 % higher in MgT group (Kolmogorov-Smirnov test, p < 0.0005) than that in control group, suggesting that the administration of MgT can promote the presence of Ca^2+^-sensitivity-related proteins in the Stratum Radiatum layer of CA1.

Furthermore, we checked the quantity of Ca^2+^-sensitivity-related proteins relative to the structure-related protein SYP (i.e. *ΣQ*_*proteins*_/*Q*_*SYP*_), and found that it was linearly correlated with *N*_*mito*_ in both control and MgT groups (Fig. 8e). After MgT supplementation, both *N*_*mito*_ and *ΣQ*_*proteins*_/*Q*_*SYP*_ increased, maintaining the linear correlation between each other (Fig. 8e). These data were also similar to the *in vitro* data (Fig. 7c and d).

Altogether these *in vivo* results were in agreement with the *in vitro* findings, and further supported our hypothesis that Mg^2+^ plays an important role in controlling mitochondrial density in branches, which in turn determines the expression of Ca^2+^-sensitivity-related proteins in terminals.

## Discussion

### Presynaptic Ca^2+^ sensitivity, intracellular Mg^2+^ and functional terminal density

In the current study, we observed that only half of presynaptic terminals were responsive to physiological pattern of input (Fig. 3a-d), which is similar to what is reported in prior studies [21, 20, 24, 63]. We found that elevating [Mg^2+^]_o_ is very effective in converting these nonfunctional terminals into functional ones (Fig. 2). Increasing [Mg^2+^]_o_ was used as a tool to elevate [Mg^2+^]_i_, resulting in an increase in the ratio of functional/nonfunctional terminals. While elevating [Mg^2+^]_o_ could lead to the increase of participation of the functional terminals during the enhancement of presynaptic plasticity via boosted NR2B-containing NMDAR-induced retrograde signaling (see our previous study [24]), here we show evidence suggesting that the change in functional terminal density is primarily due to [Mg^2+^]_i_. First, [Mg^2+^]_o_ had a bell-shape relationship with functional terminal density (Fig. 1c) and [Mg^2+^]_i_ (Fig. 1d), whereas [Mg^2+^]_o_ is positively correlated with NMDAR upregulation (see Fig. 7 of [24]). Also, [Mg^2+^]_i_ was positively correlated with functional terminal density (Fig. 2b). Second, imipramine, which can elevate [Mg^2+^]_i_ via the blockage of Mg^2+^ extrusion, temporally altered functional terminal density (Fig. 2c and f). Together, these data suggest that functional terminal density is primarily due to [Mg^2+^]_i_, which can be mediated by [Mg^2+^]_o_.

By manipulating [Mg^2+^]_o_ to affect [Mg^2+^]_i_, we revealed that very low presynaptic Ca^2+^ sensitivity was largely responsible for the presence of nonfunctional terminals (Fig. 3e and f). This low Ca^2+^ sensitivity of synaptic release machinery was caused by the insufficient quantity of terminal Ca^2+^-sensitivity-related proteins (e.g. SYT1, Rab3, RIM1, Munc13, ELKS and Syntaxin1) (Fig. 4c and d). Previous studies suggest the possible association between the lack of presynaptic cytomatrix proteins and presynaptic silencing [41, 42, 25, 43], and have extensively investigated the effects of Ca^2+^-sensitivity-related proteins (as well as their interactions) on transmitter release (see review [44, 64]) and on the underlying molecular pathways involved in the regulation of presynaptic release [16, 17, 26]. However, it remains elusive how to upregulate the expression of Ca^2+^-sensitivity-related proteins in terminals under physiological conditions. The current study showed that elevating [Mg^2+^]_i_ resulted in more functional terminals within a branch. Increased [Mg^2+^]_i_ resulted in the conversion of nonfunctional terminals into functional ones (Fig. 2c, d and f), and was associated with an increase in Ca^2+^-sensitivity-related proteins in terminals (Fig. 4c and d). These data indicate that intracellular Mg^2+^ might be a critical endogenous enhancer of terminal Ca^2+^-sensitivity-related protein quantity, and in turn, a regulator of functional terminal density. Hence, regulating [Mg^2+^]_i_ may be a valid way to control functional terminal density under physiological conditions. It should be noted that a limitation of our protein turnover analysis experiments (Fig. 5b-f) was that they did not provide direct evidence that elevated [Mg^2+^]_i_ accelerated the axonal transport of Ca^2+^-sensitivity-related proteins from soma to terminals, and such experiment should be done in future studies.

### Local energy supply, Mg^2+^ and functional terminal density

Of all organs or tissues, the brain consumes the highest energy per unit weight [65, 66]. Most of the brain's energy consumption goes into sustaining neural activity within networks, with ~80 % or more consumed by synaptic transmission (including action potentials and postsynaptic effects) [65, 66]. Therefore, the synaptic network needs to maximize the efficiency of information transmission per unit energy expended. This maximization is considered a basic principle in the design of the neural network [67]. To achieve proper neural network functionality, the level of neural activity needs to be scaled to the available energy supply. The results from the current study suggest a possible solution: let local energy supply (*E*) control the fraction of synaptic terminals that can participate in synaptic transmission (i.e. *N* ∝ *E*) (Fig. 7).

**Fig. 9:**
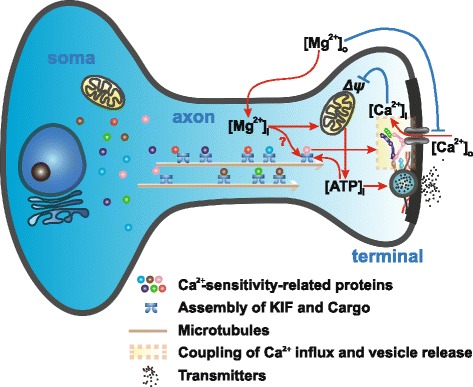
Regulation of functionality of a terminal by intracellular Mg^2+^. Schematic cartoon to illustrate the regulation of presynaptic functionality at a single terminal: ↑[Mg^2+^]_i_ ⇒ ↑Mitochondria ⇒ ↑[ATP]_i_ ⇒ ↑Axonal transport efficiency ⇒ ↑Ca^2+^-sensitivity-related proteins ⇒ ↑Ca^2+^ sensitivity ⇒ Terminal in functional state

In this study (illustrated by the schematic cartoon in Fig. 9), we show that functionality of presyanptic terminals is likely determined by the amount of Ca^2+^-sensitivity-related proteins in terminals. Interestingly, the half-life of Ca^2+^-sensitivity-related proteins (several hours) was shown to be much shorter than that of structure-related proteins (e.g. SYP) (several days) (Fig. 5e and f), indicating that their presence at the terminal is a function of transport efficiency. As protein transport in the axon is a high energy-consuming process, the status of local energy supply should directly affect the efficiency of protein transport [60]. When local energy supply is adequate, sufficient amount of Ca^2+^-sensitivity-related proteins will be transported to terminals (Fig. 7a), ensuring that most of terminals are functional in response to physiological inputs (Fig. 3c and d). However, when energy supply and subsequent protein transport efficiency are reduced, the amount of Ca^2+^-sensitivity-related proteins in terminals will drop significantly (Fig. 7a), resulting in the decline of Ca^2+^ sensitivity (Fig. 3e and f) and consequently the ability of the terminals to participate in synaptic transmission (Fig. 7). Within this construct, it is likely that over-consumption of energy will lead to down-regulation of axonal transport of Ca^2+^-sensitivity-related proteins into terminals, resulting in reduced terminal Ca^2+^ sensitivity, in order to save energy. This negative feedback control will ensure that the proportion of terminals in the functional state will always be scaled according to local energy supply (Fig. 9). The possible computational consequence of such an arrangement is that a presynaptic terminal fluctuates its ability to transfer information dependent on the extent of local energy supply: the higher the energy supply, the larger the fraction of presynaptic terminals in the functional state and the stronger the synaptic transmission during information transmission.

We showed that elevating [Mg^2+^]_o_ can concurrently enhance the number and function of mitochondria in distal branches, resulting in the increase of reserved [ATP]_i_ (Fig. 6c). Currently, we have not determined exactly how the enhancement was achieved after elevation of [Mg^2+^]_o_, but a possible mechanism is illustrated in Fig. 9. For the increase in *ΔΨ*, one possible explanation is that elevating both extra- and intracellular Mg^2+^ level, by competitively gating Ca^2+^ channels, can attenuate Ca^2+^ influx [68], which can rapidly reduce *ΔΨ* [69]. Once the *ΔΨ* of mitochondria is increased the mitochondria are more likely to undergo anterograde transport [70], which might partially account for our findings that the mitochondrial density in distal branches increased (Figs. 6–8). However, the exact mechanisms need further elucidation.

Finally, our previous work demonstrates the important role of Mg^2+^ as a positive regulator of NR2B-containing NMDARs in controlling synaptic plasticity [24]. In the current study, we revealed the molecular mechanisms of Mg^2+^ in regulating presynaptic functional terminal density (Fig. 9), and found that sufficiently high [Mg^2+^]_i_ might be essential for maintaining a high density of functional presynaptic terminals, thereby sustaining a high sensitivity of synaptic networks for information transmission. These studies provide the mechanistic understanding of how elevating brain Mg^2+^ concentration can prevent age-related memory decline in old rats [62], reverse cognitive decline in Alzheimer’s Disease (AD) model mice [71] as well as ameliorate cognitive decline in subjects with mild cognitive impairment (MCI) (GL et al. manuscript in submission).

## Methods

### Imaging and analysis

Most of the *in vitro* fluorescent or differential interference contrast (DIC) images for an area of interest (AOI) (59 μm × 59 μm) were taken at 1024 × 1024 pixels with a 0.05754 μm/pixel resolution using a 60X NA1.20 water-immersion objective on a Laser Scanning Confocal Microscopy System FV300 (Olympus) at room temperature (RT, 22–26 °C), the exceptions were clarified in the following methods. A z-stack of images was taken with 0.5-1 μm steps and compressed at maximal intensity to generate the final image, which was then processed and analyzed in Image-Pro Plus 5.0 (IPP5.0) (Media Cybernetics) and/or an open source software Fiji (Image J). For the measurements of vesicle turnover and protein expression in terminals at dendrites, the AOIs were chosen at the high-density regions of dendritic branches among the “islands” of cell bodies. The general criterion for choosing a “high-density dendritic area” was that most of area in the AOI was covered by neuronal branches. For the fluorescent colocalization experiments (described below), the images from the same AOI were aligned together and registered using the algorithms Rigid Body Registration [72] and UnwarpJ [73] to register the images and tackle the distortions in Fiji. For *in vivo* experiments, the Electron Microscopy (EM) images and immunofluorescent images of ultrathin brain slices were described below.

### Hippocampal neuron cultures

High-density primary cultures of hippocampal CA3-CA1 pyramidal neurons of neonatal *Sprague–Dawley* rats (<24 hr) were used in the current study, as described before [24]. The neurons were cultured on 1# coverslips (8 mm × 8 mm) for 14–28 div before use. [Mg^2+^]_o_ in culture medium was adjusted to different concentrations according to experimental designs. In each experiment, sister cultures from the same batch were always used for treatment and control conditions on the same day, and the experimental results were repeated using several different batches.

### Vesicle turnover detection

FM dyes were used to visualize the vesicle turnover of presynaptic terminals as previously described [24]. In the current study, 10 μM FM1-43 or 20 μM FM4-64 (Biotium) was loaded under different patterns of field stimulations, such as 5AP bursts (5 APs at 100Hz for each burst, 6 bursts (30 APs totally) with 10 s inter burst interval were given) and “maximal stimulation” (600 APs at 10 Hz) [54]. Releasable fluorescence in the AOI (*ΔF*) was obtained from the difference of loading (*F*_*1*_) and unloading (*F*_*2*_) images, i.e. *ΔF* = *F*_*1*_ – *F*_*2*_ (see Fig. 1a). For Fig. 3c, the loading/unloading procedures were performed under 5AP bursts and 600AP stimulation at the same area of network successively. Subsequently, the *ΔF*_*5AP*_ and *ΔF*_*600AP*_ images were registered to colocalize the vesicle turnover for these two stimulating protocols for individual terminals.

### Functional terminal density measurement

The functional terminal density (*N*_*5AP*_) at a dendritic branch was defined as the number of 5AP bursting stimulation induced FM(+) puncta (*#FM*_*5AP*_) per unit area of dendritic branch (*A*), *N*_*5AP*_ = *#FM*_*5AP*_*/A*. Retrograde immunofluorescence (IF) staining of MAP2 (as described below), which specifically presents in dendritic skeleton, was used to label dendritic area and *A* was calculated from MAP2(+) area. To limit the error of *A* introduced by the immunoreactivity of MAP2(+), the staining and imaging parameters were rigorously controlled in different batches of IF and the grey-scale histograms of MAP2(+) images were equalized before measuring *A*. Here, to ensure the equalized MAP2(+) marked area could reflect the real morphology of dendrites, the parameters of histogram equalization were set based on the comparison of MAP2(+) and DIC images. For most experiments, the average *N*_*5AP*_ of an AOI was calculated from the total number of FM(+) puncta divided by total MAP2(+) area to reduce the sampling error. Then the mean *N*_*5AP*_ of all AOIs (1–5 AOIs/coverslip) was used to represent the average *N*_*5AP*_ in a coverslip; for each statistical data point, several coverslips were used and the mean ± SEM of all coverslips was represented (see Figure legends). For Fig. 3b and d, the FM(+) puncta density induced by 600AP stimulation was also calculated and presented in the same way as described above. Exceptionally, for Fig. 2b, each point represented the *N*_*5AP*_ at a single segment of dendritic branch. In general, in an AOI (59 μm × 59 μm, with varying dendritic density) 351–1598 presynaptic functional terminals could be detected by FM dyes for different experimental groups under 5AP bursting stimulation.

### Immunofluorescence *in vitro*

The neurons were fixed in 1X PBS (pH 7.4) containing 1 % Paraformaldehyde (E.M.S.), 0.01 % Glutaraldehyde (Alfa Aesar) and 4 % Sucrose (Amresco) for 1 hr RT. The fixatives were gently washed several times in Tris buffer (pH 7.6), which contained (mM): Tris Base 25, Tris–HCl 25, NaCl 150 (Amresco), and was filtered through 0.22 μm film before use. The coverslips were then permeablized and blocked in freshly prepared blocking solution (using the Tris buffer), which contained 1 % (w/v) BSA (Amresco) and 0.1 % (w/v) Saponin (Sigma) for 1.5 hr RT. Primary antibodies were incubated overnight at 4 °C and probed with CF-dye conjugated secondary antibodies (Biotium) for 1 hr RT. Z-stack images (with 0.5 μm step in z-direction) were taken immediately on FV300, and processed as described above. Specifically, images from 236 μm × 236 μm regions were taken at 2048 × 2048 pixels for Fig. 5b.

The following primary and secondary antibodies were used (name, catalogue number, company and dilution): anti-MAP2 (188004, SYSY or NB300-213, Novus) 1:200–1:400; Synaptophysin (SYP) (MAB5258, Millipore or 101 004 SYSY) 1:400; VGLUT1 (AB5905, Millipore) 1:300; VGAT (AB5062, Millipore) 1:200; Bassoon (141 003, SYSY) 1:200; Synapsin1 (AB1543, Millipore) 1:400; Ca_v_2.2 (AB5154, Millipore) 1:200; Ca_v_2.1 (152 103, SYSY) 1:200; Synaptotagmin1 (SYT1) (105 011 or 105 003, SYSY) 1:200; Rab3a (107 111 or 107 102, SYSY) 1:200; RIM1 (140 013 or 140 003, SYSY) 1:200; Munc13-1 (126 103, SYSY) 1:200; ERC1b2 (ELKS) (143 003, SYSY) 1:200; Syntaxin1 (110 011, SYSY) 1:100. CF-dye conjugated secondary antibodies: CF488A 1:400, CF555 1:100–1:200 and CF640R 1:100–1:200 (Biotium).

### *In vitro* protein immunofluorescence quantifications

For Fig. 3b, the average density of immunoreactive puncta of a given AOI was calculated from the total puncta number divided by the total MAP2(+) area; the values from several AOIs were averaged to represent the average puncta density in a coverslip; for each data bar, the mean ± SEM of these coverslips were presented. For Figs. 4d, 5d-f, the total fluorescence intensity of all immunoreactive puncta in an AOI was divided by the total MAP2(+) area to get the average immunoreactivity per unit area of dendrites in the AOI; several AOIs were measured from a coverslip and the mean of these AOIs was calculated to represent the average immunoreactivity per unit area of dendrite in the coverslip; for each data point, several coverslips were used and the mean ± SEM of these coverslips were presented. For Fig. 5c and d, the mean fluorescence intensity per pixel in each cell body was measured (the somatic area of each cell body was measured from DIC image) and then the mean ± SEM of all the cell bodies were presented. For Fig. 7b-e, the average protein immunoreactivity of an AOI was defined as the total fluorescence of a protein per unit area of MAP2(+) marked dendrites (as described above); 9 AOIs were selected from a low [Mg^2+^]_o_ (Ctrl 0.8) coverslip or a high [Mg^2+^]_o_ (1.2 4hr) coverslip respectively (these two coverslips were sister cultures from the same culturing dish); for each protein, the average protein immunoreactivity values were normalized to the mean of the 9 AOIs of Ctrl 0.8 group. We defined the normalized immunoreactivity per unit area of dendrites as *Q* index, then *ΣQ*_*proteins*_ = *Q*_*SYT1*_ + *Q*_*Rab3a*_ + *Q*_*RIM1*_ + *Q*_*Munc13*_ + *Q*_*ELKS*_ + *Q*_*Syntaxin1*_.

### Intracellular Mg^2+^ measurement

The mature cultures were incubated in Tyrode solution with 2 μM Magnesium Green-AM (MgGrn, an intracellular Mg^2+^ indicator, Invitrogen) for 30 min and washed out for 20 min; both at 37 °C. Fluorescent images of several AOIs in each coverslip were taken on FV300 at RT. In the current study, we measured the MgGrn fluorescence at basal condition (without eliciting any AP stimulus) (Fig. 1a) to eliminate any effects caused by AP-induced intracellular Ca^2+^ fluctuations. Since the intracellular Ca^2+^ concentration ([Ca^2+^]_i_) at basal condition was usually < 100 nM, the contribution of Ca^2+^ to MgGrn fluorescence was very small [74]. Thus, the fluorescent intensity of MgGrn could be considered proportional to [Mg^2+^]_i_. Since the measured MgGrn fluorescence was also dependent on the local volume of branches in network, we built a physical model to describe this phenomenon and derived the calculating formula that corrected the effect of varying local volumes.

At basal condition, the total fluorescence in a branch was positively proportional to the total amount of free Mg^2+^ ions in cytoplasm. We assumed a cylindrical shape of each neuronal branch (Additional file 1: Figure S1. A); thus we could deduce the following relationship:

$$ flu{o}_{total}=\underset{V\hbox{'}}{\iiint }F\left(x,y,z\right)dv=\underset{V\hbox{'}}{\iiint }F\left(x,y,z\right) dxdydz $$

Where *fluo*_*total*_ was the total fluorescence, *F*(*x, y, z*) was the spatial distribution function of fluorescence, *dv* was the differential volume at the point (*x, y, z*), *V’* represented the volume of fluorescence detectable space.

Then we derived the relationship as follows based on the assumption that [Mg^2+^]_i_ was proportional to integral fluorescence per unit volume of a neuronal branch:

$$ {\left[\mathrm{M}{\mathrm{g}}^{2+}\right]}_i\propto \frac{{\displaystyle \underset{V\hbox{'}}{\iiint }F\left(x,y,z\right)dxdydz}}{V} $$

Where *V* was the volume of the branch.

From Additional file 1: Figure S1. A and B we could further derive that:

$$ {\left[\mathrm{M}{\mathrm{g}}^{2+}\right]}_i\propto \frac{{\displaystyle \underset{-h}{\overset{h}{\int }}dz}{\displaystyle \underset{d}{\int }dy\underset{L}{\int }F\left(x,y,z\right)dx}}{\frac{1}{4}\pi {d}^2L} $$

Where [−*h, h*] was the range in *z*-direction, where fluorescence was detectable, *L, d* were the length (in *x*-direction) and width (in *y*-direction) of the maximal projection of fluorescence on *xy*-plane (Additional file 1: Figure S1. B), which were roughly equal to the length and diameter of the branch when the threshold of fluorescence during segmentation was set properly. Thus the branch volume *V* was approximately equal to *πd*^*2*^*L/4*.

We set *θ* (μm/pixel) as the resolution in *x-* and *y-*direction, which was 0.05754 μm/pixel in our experiments, and set *ρ* as the resolution in *z-*direction, which was equal to the scanning step of z-stack, i.e. *ρ* = 1 μm. Thus *L = lθ*, *d = mθ*, *2h = nρ*, where *l, m* were the pixels of the branch in *x, y* directions, *n* was the number of images in the stack. Then we got:

$$ {\left[\mathrm{M}{\mathrm{g}}^{2+}\right]}_i\propto \frac{4}{\pi {\theta}^2d}\cdot \frac{{\displaystyle \underset{-h}{\overset{h}{\int }}dz}{\displaystyle \underset{d}{\int }dy}{\displaystyle \underset{L}{\int }F}\left(x,y,z\right)dx}{ml} $$

By approximate numerical integration, we derived:

$$ {\left[\mathrm{M}{\mathrm{g}}^{2+}\right]}_i\propto \frac{4}{\pi {\theta}^2d}\cdot \frac{{\displaystyle \sum_{z=-\frac{1}{2}n\rho}^{\frac{1}{2}n\rho}\sum_{y=0}^{m\theta}\sum_{x=0}^{l\theta }F\left(x,y,z\right)}}{ml}=\frac{4}{\pi {\theta}^2d}\cdot \sum_{z=-\frac{1}{2}n\rho}^{\frac{1}{2}n\rho}\frac{\kern0.1em }{F(z)} $$

Where $\frac{\kern0.1em }{F(z)}$ was the mean fluorescence per pixel in the region of the branch in the image (whose coordinate was *z*) in z-stack (Additional file 1: Figure S1. B).

Since we observed that in the range of [−*nρ*/2, *nρ*/2], $\frac{\kern0.1em }{F(z)}$ exhibited well Gaussian distribution (Additional file 1: Figure S1. C), and the normalized distribution curve was the same among different branches (Additional file 1: Figure S1. C), then we knew $\frac{\kern0.1em }{\varSigma F(z)}$ was linearly proportional to $\frac{\kern0.1em }{F{(z)}_{\max }}$ according to the properties of Gaussian curve, thus:

$$ {\left[\mathrm{M}{\mathrm{g}}^{2+}\right]}_i\propto \frac{4}{\pi {\theta}^2d}\cdot \overline{{F{(z)}_{\max }}\raisebox{9.2pt}{}} $$

And we could further simplify this formula as follows:

$$ {\left[\mathrm{M}{\mathrm{g}}^{2+}\right]}_i\propto \frac{\overline{{F{(z)}_{max}}\raisebox{9.2pt}{}}}{d} $$

Where $\frac{\kern0.1em }{F{(z)}_{\max }}$ was the mean intensity per pixel in the area of a branch in the compressed image of the stack (the compressed image was achieve by maximal *z*-projection of the stack, as described above).

From the formula, we knew that [Mg^2+^]_i_ was approximately positively proportional to $\frac{\kern0.1em }{F{(z)}_{\max }}$ but negatively proportional to the diameter of branch, consistent with the experimental observations (Additional file 1: Figure S1. D and E).

In the experiments, given a randomly selected AOI of dendritic area (the image was obtained as described above), 50–100 branches were randomly selected from each AOI and the mean intensity of fluorescence (i.e. $\frac{\kern0.1em }{F{(z)}_{\max }}$) of each branch was measured. Meanwhile, the diameter of each branch (*d*) was measured from the DIC image, then the $\frac{\kern0.1em }{F{(z)}_{\max }/d}$ was calculated to represent the [Mg^2+^]_i_ in the branch. The mean value of these 50–100 branches was calculated to represent the level of [Mg^2+^]_i_ in the AOI. For each coverslip, several AOIs were measured and the mean value of these AOIs was calculated to represent the average level of [Mg^2+^]_i_ in the coverslip. For each data point, several coverslips were used. For each statistical data point, the mean ± SEM of all coverslips was presented. Specifically for Fig. 2b, each data point represented the [Mg^2+^]_i_ (MgGrn fluo. a.u.) value in an individual branch.

### Mitochondrial status assessment

For Fig. 7a, mitochondria were marked with 40 nM Mitoview633 (Biotium) for 15 min and then washed out in blank medium for 15 min, both at 37 °C. For Fig. 6a, the membrane potential-sensitive fluorescent dye JC-1 (1 μM) (Invitrogen) was used. JC-1 was incubated for 15 min followed by a 15 min washout at 37 °C. Fluorescent images were taken on FV300 confocal. Mitochondrial membrane potential (*ΔΨ*) of each mitochondrion was estimated by the ratio of fluorescence of aggregate versus monomer form of JC-1, i.e. *F*_*aggr*_*/F*_*mono*_ [61]. JC-1 was excited by 488 nm laser and the emission spectra was collected at 510–575 nm and > 575 nm at the same time separately. Then we calculated the ratio of fluorescence at > 575 nm versus at 510–575 nm (*F*_*aggr*_*/F*_*mono*_) for each individual mitochondrial punctum to represent the *ΔΨ* of that mitochondrion. To estimate the valid range of *F*_*aggr*_*/F*_*mono*_, we measured *F*_*aggr*_*/F*_*mono*_ of individual mitochondria before and after administering 5 μM FCCP 5 min at the same AOI. The mean *F*_*aggr*_*/F*_*mono*_ of all the mitochondria (1.21 ± 0.02, n = 942 mitochondria) was lowered to 0.26 ± 0.002 (the minimal value of *F*_*aggr*_*/F*_*mono*_). Thus, we considered the *F*_*aggr*_*/F*_*mono*_ value of a mitochondrion to be meaningful only if it was > 0.26 in our experiments, and the puncta with *F*_*aggr*_*/F*_*mono*_ < 0.26 (17/942 mitochondria in this experiment, i.e. ~2 %) were excluded in statistics. *F*_*aggr*_*/F*_*mono*_ was calculated for each individual meaningful punctum and the mean value of population was calculated from all the puncta to represent the general *ΔΨ* in this AOI. 3–5 AOIs were measured for each coverslip and the mean of the AOIs were calculated to represent the average *ΔΨ* of the coverslip. For each bar or point in Fig. 6, several coverslips were used (described in legends). We also quantified the density of mitochondria (*N*_*mito*_) by counting the number of JC-1-aggregate(+) fluorescent puncta (Fig. 6) or MitoView633(+) puncta (Fig. 7) per area of branches. We observed no difference in *N*_*mito*_ measurement between the two markers.

### Co-staining of multiple fluorescent markers

For Fig. 3a, FM1-43 staining was performed and images were taken on confocal. Then retrograde IF was performed at the same AOI. For Fig. 7a, MitoView633 was used to mark mitochondria, images were taken, and then FM1-43 staining was performed and images were taken. For the combination of FM and IF imaging, a new strategy of retrograde IF was performed for several rounds to label multiple presynaptic proteins at a given AOI (HZ and GL unpublished method: Single Synapse Analysis by FM1-43 and Immunofluorescence Imaging Array, which we named SAFIA). For each round, 2–3 antibodies were stained and probed by fluorophore-conjugated secondary antibodies (as described above), and then both the DIC and the fluorescent images were taken immediately. After taking all images, primary and secondary antibodies were completely eluted (data not shown) by a stripping buffer containing 0.2 M NaOH and 0.015 % (w/v) SDS (Amresco) in deionized water for 20 min RT, twice, and then washed out gently and thoroughly in Tris buffer (described above) for more than 1 hr RT (to make sure no residual SDS was left). Then the IF of other 2–3 primary antibodies at the same AOI was performed as described above and images of these AOIs were taken. DIC image was used as landmark of each AOI. All the fluorescent images were aligned and registered in Fiji and analyzed as described above. In the first round of IF in the SAFIA experiments, 300 μM ADVASEP-7 (Biotium) was added into the blocking solution during the permeablization procedure to quench the residual FM-dye in the membrane and to ensure that the background introduced by FM-dye was as low as possible in the following IF experiments (data not shown). For Figs. 1b and 2a, [Mg^2+^]_i_ was stained with MgGrn, images were taken at basal condition, and then the FM4-64 staining was performed. All the procedures were described separately as aforementioned.

### Western blot

[Mg^2+^]_o_ was elevated from 0.8 to 1.2 mM in mature sister cultures (14–28 div) for 6 hr. Proteins were extracted from the cultures and then resolved on Polyacrylamide gels. Proteins were transferred from gels to PVDF membrane and probed with the following antibodies: anti-SYP (Millipore) 1:50,000, SYT1 1:5,000, Rab3a 1:500–1,000, RIM1 1:800, Munc13-1 1:400, ERC1b2 (ELKS) 1:1,000, Syntaxin1 1:500–1,000 (SYSY), GAPDH 1:1,000 (CST). The other procedures were the same as previously described [62]. For the analysis, digital images were quantified using IPP5.0, and the level of each protein was normalized by the level of GAPDH in the same lane. The sample from each coverslip was resolved individually and repeated for 3 times, and then the average value of the 3 times was calculated to represent the protein level in the coverslip. For each bar in Fig. 5a, the mean ± SEM of coverslips was presented.

### ATP measurement

Cultured neurons were quickly detached from a coverslip with 500 μL boiling extraction solution [75] containing 40 mM HEPES (pH 7.8) and 4 mM MgSO_4_. The solution was repeatedly pipetted to ensure complete lysis. The lysed neurons were immediately transferred into a centrifugal tube and centrifuged at 12,000 rpm at 4 °C for 5 min. For each coverslip, total ATP content of supernatant was immediately determined using a Luciferin-luciferase ATP Assay Kit (Invitrogen). For each data point in Figs. 6c, 7e and f, the mean ± SEM of coverslips was presented.

### Electron microscopy and immunofluorescence *in vivo*

Mature *Sprague–Dawley* rats (male, 16 months old) were fed Magnesium L-Threonate (MgT; dosage as previously described) (Magceutics Inc.) in their water for 8 months [62]. Control (n = 10) and MgT-supplemented (n = 11) rats (24 months old) were anesthetized with 4 % Benbarbitol (0.2 mL/100 g body weight), and sacrificed by transcardio-perfusion of 1 % Paraformaldehyde (E.M.S. or Ted Pella), 0.01 % Glutaraldehyde (Alfa Aesar) and 0.05 % Picric Acid (Sigma) in 1X PBS (Gibco) (pH 7.4, pre-cooled at 4°C). Each brain was dissected, the hippocampus was coronally sliced on a vibratome (VT1000S Vibratome, Leica) into 50 μm sequential sections, and slices were stored in fixative solution at 4 °C for 24 hr. The Hippocampus CA1 Stratum Radiatum region from the neighbor sections was cut off and dissected into < 1 mm^2^ square blocks which were then used for electron microscopy (EM) and IF separately. The blocks were rinsed in 1X PBS (pH 7.4) for 1 hr and 1X Maleate Acid (Sigma) buffer (pH 6.0) for 1 hr to wash out the fixatives.

For the blocks prepared for EM, they were post-fixed in 1 % Osmium Tetraoxide (Ted Pella) and 1.5 % Potassium Ferricyanide (Sigma) in MB (pH 6.0) for 1 hr, and stained with 3 % Uranyl Acetate (Ted Pella) for 1 hr. Then the blocks were dehydrated with 50, 70, 80, 95 %, 100 %, and 100 % Ethanol sequentially, for 15 min each. Following dehydration, blocks were placed in 100 % Propyleneoxide (Sigma) for 10 min twice. All the procedures above were performed on ice. Then blocks were infiltrated with 50 % Propyleneoxide + 50 % SPI-PON 812 resin (SPI-CHEM) for 60 min RT, 100 % resin for 24 hr at 4 °C, and subsequently transferred into embedment molds fulfilled with pure resin and placed in 60 °C oven for polymerization for 24 hr. 70 nm ultrathin slices were cut and then stained with 3 % Uranyl Acetate and 0.4 % (w/v) Lead Citrate, sequentially. Images were taken on Transmission (Hitachi H-7650) (80 kV, 1k × 1k pixels, 25kX, 2 nm/pixel) or Scanning EM (Supra55, Zeiss) (10 kV, 8k × 8k pixels, 2 nm/pixel, 15 μs/pixel). For the SEM, ultrathin slices were mounted on silicon chips, which can enhance the electro-conductivity dramatically (even without carbon coating). The density of mitochondria in an EM image was defined as #mitochondria/area. For each rat, the density of mitochondria was calculated from the mean density of 67–96 TEM images and 5 SEM images (the density measured in TEM images was the same as that measured in SEM images).

For the blocks prepared for IF, they were treated with 1 % Tannic Acid (Sigma) for 1hr, 1 % Uranyl Acetate in Maleate Acid buffer (pH 6.0) for 1hr, and then dehydrated with 50, 70, 80, 95, 100 %, and 100 % Ethanol containing 1 % PPD (Sigma) sequentially, for 15 min each. Mixture of Ethanol and LR White resin (E.M.S.) with the ratios 2:1, 1:1, 2:1 and pure resin were used to infiltrate the tissue blocks, each for 1 hr, then replaced by 100 % LR White for 24 hr. All the above procedures were performed on ice. The blocks were placed in capsules filled with LR White resin and put in 55 °C oven for polymerization for 48 hr. 70 nm ultrathin slices were cut and mounted on coverslips. The IF was performed according to Array Tomography technique [76]. SYP, VGLUT1, SYT1, Rab3a, RIM1, Munc13-1, ELKS and Syntaxin1 were stained in the ultrathin slices and fluorescent images of each protein were taken at 1024 × 1024 resolution with 0.03836 μm/pixel resolution using a 60X objective (UPlanSApo 60XW N.A. 1.20) on FV300. For each protein, the total fluorescence (*F*) of all the puncta per area of image was measured to represent the quantity of that protein. For each rat, *F* values from 5–7 tissue blocks were averaged to represent the protein quantity in that rat (*F*_*rat*_). We normalized *F*_*rat*_ of each protein by the mean value of all rats in control group to get the normalized quantity *Q*. To estimate the total quantity of the Ca^2+^-sensitivity-related proteins, we summated the *Q* of the 6 proteins in the same rat together to represent the total quantity in that rat (*ΣQ*_*proteins*_), *ΣQ*_*proteins*_ = *Q*_*SYT1*_ + *Q*_*Rab3a*_ + *Q*_*RIM1*_ + *Q*_*Munc13*_ + *Q*_*ELKS*_ + *Q*_*Syntaxin1*_.

### Experimental animals

All the rats involved in this paper were purchased from Vital River Laboratory (Beijing). All the animal experiments were approved by Tsinghua University Committees on Animal Care.

### Statistics analysis

Data are shown as mean ± SEM. Statistical significance is considered as *p* < 0.05. Two-tailed Student-t or Kolmogorov-Smirnov test is used.

## Additional file

Additional file 1: Figure S1. Modeling the measurement of [Mg^2+^]_i_ in a segment of branch by MgGrn fluorescence. (A) A segment of branch was modeled as a cylinder characterized by diameter (*d*) and length (*L*). (B) Z-stack of MgGrn fluorescent images was taken in the range of –*h* to *h* at *z*-axis. $\frac{\kern0.1em }{F(z)}$ represented the mean fluorescent intensity per pixel within a branch area from each layer in the z-stack (left). Theoretically, $\frac{\kern0.1em }{F(z)}$ should exhibited a Gaussian distribution along z-axis (right). (C) Normalized $\frac{\kern0.1em }{F(z)}$ values (normalized to maximum) from z-stacks of 5 representative branches (No.1-5) exhibited well Gaussian distributions with almost the same shape in parallel experiments (Gaussian curve fitting). (D) In the maximal z-projection of the stack, the mean intensity of individual branches ($\frac{\kern0.1em }{F{(z)}_{\max }}$) showed a linear correlation with diameter (*d*). (E) After correction, the value $\frac{\kern0.1em }{F{(z)}_{\max }/d}$ was not correlated with diameter in different branches.

## Abbreviations

AMPA: α-amino-3-hydroxy-5-methyl-4-isoxazole-propionic acid
AOI: Area of interest
AP: Action potential
ATP: Adenosine triphosphate
BSA: Bovine serum albumin
Ca^2+^: Calcium ion
CA3-CA1: *Cornus ammonis* 3, *cornus ammonis* 1 region in hippocampus
Colch: Colchicine
*ΔΨ*: Mitochondrial membrane potential
DIC: Differential interference contrast
EM: Electron microscopy
*F*: Fluorescent image or intensity of fluorescence
GAPDH: Glyceraldehyde-3-phosphate dehydrogenase
IF: Immunofluorescence
IM: Imipramine
MAP2: Microtubule associated protein 2
Mg^2+^: Magnesium ion
MgGrn: Magnesium Green^TM^ AM-ester
MgT: Magnesium-L-Threonate
*N*_*5AP*_: Functional terminal density responding to 5AP bursts stimulation
NR2B: N-methyl D-aspartate receptor subtype 2B
NMDAR: N-methyl-D-aspartate receptor
*N*_*mito*_: Density of mitochondria
PBS: Phosphate buffered saline
*Pr*: Probability of presynaptic vesicle release
PVDF: Polyvinylidene difluoride
*Q*: Protein quantity, which is represented by fluorescent intensity of immunoreactive puncta
SYP: Synaptophysin
SYT1: Synaptotagmin1
VGAT: Vesicle gamma amino butyric acid transporter
VGLUT1: Vesicle glutamate transporter 1
[ATP]_i_: Intracellular ATP concentration
[Mg^2+^]_CSF_: Mg^2+^ concentration in cerebrospinal fluid
[Mg^2+^]_i_: Intracellular Mg^2+^ concentration
[Mg^2+^]_o_: Extracellular Mg^2+^ concentration
*ΣQ*_*proteins*_: Total quantity of fluorescent intensity of immunoreactive Ca^2+^-sensitivity-related proteins tested

## References

[1] Abbott LF, Regehr WG (2004). Synaptic computation. Nature.

[2] Hanse E, Seth H, Riebe I. AMPA-silent synapses in brain development and pathology. Nature Reviews Neuroscience. 2013;14(12):839–50. doi:10.1038/Nrn3642. 10.1038/nrn3642 24201185

[3] Rumpel S, Kattenstroth G, Gottmann K (2004). Silent synapses in the immature visual cortex: Layer-specific developmental regulation. J Neurophysiol.

[4] Bottjer SW (2005). Silent synapses in a thalamo-cortical circuit necessary for song learning in zebra finches. J Neurophysiol.

[5] Li P, Zhuo M (1998). Silent glutamatergic synapses and nociception in mammalian spinal cord. Nature.

[6] Isaac JT, Nicoll RA, Malenka RC (1999). Silent glutamatergic synapses in the mammalian brain. Can J Physiol Pharmacol.

[7] Sametsky EA, Disterhoft JF, Geinisman Y, Nicholson DA (2010). Synaptic strength and postsynaptically silent synapses through advanced aging in rat hippocampal CA1 pyramidal neurons. Neurobiol Aging.

[8] Kerchner GA, Li P, Zhuo M (1999). Speaking out of turn: A role for silent synapses in pain. Iubmb Life.

[9] Hogins J, Crawford DC, Jiang X, Mennerick S (2011). Presynaptic silencing is an endogenous neuroprotectant during excitotoxic insults. Neurobiol Dis.

[10] Zhou C, Lippman JJ, Sun H, Jensen FE (2011). Hypoxia-induced neonatal seizures diminish silent synapses and long-term potentiation in hippocampal CA1 neurons. J Neurosci.

[11] van Spronsen M, Hoogenraad CC. Synapse Pathology in Psychiatric and Neurologic Disease. Curr Neurol Neurosci. 2010;10(3):207–14. doi:10.1007/S11910-010-0104-8. 10.1007/s11910-010-0104-8 PMC2857788 20425036

[12] Huang YH, Schluter OM, Dong Y. Silent Synapses Speak Up: Updates of the Neural Rejuvenation Hypothesis of Drug Addiction. The Neuroscientist : a review journal bringing neurobiology, neurology and psychiatry. 2015. doi:10.1177/1073858415579405. 10.1177/1073858415579405 PMC4675132 25829364

[13] Isaac JT, Nicoll RA, Malenka RC (1995). Evidence for silent synapses: implications for the expression of LTP. Neuron.

[14] Liao D, Hessler NA, Malinow R (1995). Activation of postsynaptically silent synapses during pairing-induced LTP in CA1 region of hippocampal slice. Nature.

[15] Kerchner GA, Nicoll RA (2008). Silent synapses and the emergence of a postsynaptic mechanism for LTP. Nat Rev Neurosci.

[16] Crawford DC, Mennerick S. Presynaptically Silent Synapses: Dormancy and Awakening of Presynaptic Vesicle Release. The Neuroscientist : a review journal bringing neurobiology, neurology and psychiatry. 2012;18(3):216–23. doi:10.1177/1073858411418525. 10.1177/1073858411418525 PMC3951726 21908849

[17] Borst JGG. The low synaptic release probability in vivo. Trends in neurosciences. 2010;33(6):259–66. Doi: 10.1016/J.Tins.2010.03.003. 10.1016/j.tins.2010.03.003 20371122

[18] Wojtowicz JM, Smith BR, Atwood HL. Activity-Dependent Recruitment of Silent Synapses. Ann Ny Acad Sci. 1991;627:169–79. doi:10.1111/J.1749-6632.1991.Tb25922.X. 10.1111/j.1749-6632.1991.tb25922.x 1652913

[19] Neale EA, Nelson PG, Macdonald RL, Christian CN, Bowers LM (1983). Synaptic-Interactions between Mammalian Central Neurons in Cell-Culture.3. Morphophysiological Correlates of Quantal Synaptic Transmission. J Neurophysiol.

[20] Murthy VN, Sejnowski TJ, Stevens CF (1997). Heterogeneous release properties of visualized individual hippocampal synapses. Neuron.

[21] Ma L, Zablow L, Kandel ER, Siegelbaum SA (1999). Cyclic AMP induces functional presynaptic boutons in hippocampal CA3-CA1 neuronal cultures. Nat Neurosci.

[22] Moulder KL, Meeks JP, Shute AA, Hamilton CK, de Erausquin G, Mennerick S (2004). Plastic elimination of functional glutamate release sites by depolarization. Neuron.

[23] Moulder KL, Jiang X, Taylor AA, Olney JW, Mennerick S (2006). Physiological activity depresses synaptic function through an effect on vesicle priming. J Neurosci.

[24] Slutsky I, Sadeghpour S, Li B, Liu G (2004). Neuron.

[25] Lazarevic V, Schone C, Heine M, Gundelfinger ED, Fejtova A (2011). Extensive remodeling of the presynaptic cytomatrix upon homeostatic adaptation to network activity silencing. J Neurosci.

[26] de Jong AP, Verhage M (2009). Curr Opin Neurobiol.

[27] Tong G, Malenka RC, Nicoll RA (1996). Long-term potentiation in cultures of single hippocampal granule cells: a presynaptic form of plasticity. Neuron.

[28] Moulder KL, Jiang X, Chang C, Taylor AA, Benz AM, Conti AC (2008). A specific role for Ca2+−dependent adenylyl cyclases in recovery from adaptive presynaptic silencing. J Neurosci.

[29] Cousin MA, Evans GJ (2011). Activation of silent and weak synapses by cAMP-dependent protein kinase in cultured cerebellar granule neurons. J Physiol.

[30] Malenka RC, Madison DV, Nicoll RA (1986). Potentiation of synaptic transmission in the hippocampus by phorbol esters. Nature.

[31] Parfitt KD, Madison DV (1993). Phorbol esters enhance synaptic transmission by a presynaptic, calcium-dependent mechanism in rat hippocampus. J Physiol.

[32] Chang CY, Jiang X, Moulder KL, Mennerick S (2010). Rapid activation of dormant presynaptic terminals by phorbol esters. J Neurosci.

[33] Kim SH, Ryan TA (2010). CDK5 Serves as a Major Control Point in Neurotransmitter Release. Neuron.

[34] Tomizawa K, Ohta J, Matsushita M, Moriwaki A, Li ST, Takei K (2002). Cdk5/p35 regulates neurotransmitter release through phosphorylation and downregulation of P/Q-type voltage-dependent calcium channel activity. J Neurosci.

[35] Yan Z, Chi P, Bibb JA, Ryan TA, Greengard P (2002). Roscovitine: a novel regulator of P/Q-type calcium channels and transmitter release in central neurons. J Physiol Lond.

[36] Heifets BD, Castillo PE (2009). Endocannabinoid signaling and long-term synaptic plasticity. Annu Rev Phys Chem.

[37] Petersen SA, Fetter RD, Noordermeer JN, Goodman CS, DiAntonio A (1997). Genetic analysis of glutamate receptors in Drosophila reveals a retrograde signal regulating presynaptic transmitter release. Neuron.

[38] Kano M, Ohno-Shosaku T, Maejima T (2002). Retrograde signaling at central synapses via endogenous cannabinoids. Mol Psychiatr.

[39] Futai K, Kim MJ, Hashikawa T, Scheiffele P, Sheng M, Hayashi Y (2007). Retrograde modulation of presynaptic release probability through signaling mediated by PSD-95-neuroligin. Neurosci.

[40] Jakawich SK, Nasser HB, Strong MJ, McCartney AJ, Perez AS, Rakesh N (2010). Local presynaptic activity gates homeostatic changes in presynaptic function driven by dendritic BDNF synthesis. Neuron.

[41] Ding M, Chao D, Wang G, Shen K (2007). Spatial regulation of an E3 ubiquitin ligase directs selective synapse elimination. Science.

[42] Jiang X, Litkowski PE, Taylor AA, Lin Y, Snider BJ, Moulder KL (2010). A role for the ubiquitin-proteasome system in activity-dependent presynaptic silencing. J Neurosci.

[43] Yao I, Takagi H, Ageta H, Kahyo T, Sato S, Hatanaka K (2007). SCRAPPER-dependent ubiquitination of active zone protein RIM1 regulates synaptic vesicle release. Cell.

[44] Sudhof TC (2012). The presynaptic active zone. Neuron.

[45] Rhee JS, Betz A, Pyott S, Reim K, Varoqueaux F, Augustin I (2002). Beta phorbol ester- and diacylglycerol-induced augmentation of transmitter release is mediated by Munc13s and not by PKCs. Cell.

[46] Leinekugel X, Khazipov R, Cannon R, Hirase H, Ben-Ari Y, Buzsaki G (2002). Correlated bursts of activity in the neonatal hippocampus in vivo. Science.

[47] Lisman JE (1997). Bursts as a unit of neural information: making unreliable synapses reliable. Trends Neurosci.

[48] Salinas E, Sejnowski TJ (2001). Correlated neuronal activity and the flow of neural information. Nat Rev Neurosci.

[49] Buzsaki G (2002). Theta oscillations in the hippocampus. Neuron.

[50] Ranck JB (1973). Studies on single neurons in dorsal hippocampal formation and septum in unrestrained rats. I. Behavioral correlates and firing repertoires. Exp Neurol.

[51] Larson J, Wong D, Lynch G (1986). Patterned stimulation at the theta frequency is optimal for the induction of hippocampal long-term potentiation. Brain Res.

[52] Bliss TV, Collingridge GL (1993). A synaptic model of memory: long-term potentiation in the hippocampus. Nature.

[53] Guther T, Vormann J, Forster R (1984). Regulation of intracellular magnesium by Mg2+ efflux. Biochem Biophys Res Commun.

[54] Murthy VN, Stevens CF (1999). Reversal of synaptic vesicle docking at central synapses. Nat Neurosci.

[55] Del Castillo J, Katz B (1954). The Effect of Magnesium on the Activity of Motor Nerve Endings. J Physiol Lond.

[56] Sheng J, He L, Zheng H, Xue L, Luo F, Shin W (2012). Calcium-channel number critically influences synaptic strength and plasticity at the active zone. Nat Neurosci.

[57] Wu LG, Saggau P (1994). Pharmacological identification of two types of presynaptic voltage-dependent calcium channels at CA3-CA1 synapses of the hippocampus. J Neurosci.

[58] Geppert M, Goda Y, Hammer RE, Li C, Rosahl TW, Stevens CF (1994). Synaptotagmin-I - a Major Ca2+ Sensor for Transmitter Release at a Central Synapse. Cell.

[59] Karlsson JO, Sjostrand J (1970). Transport of neurotubular protein and the effect of colchicine on axonal transport. Acta Physiol Scand Suppl.

[60] Nitta R, Okada Y, Hirokawa N (2008). Structural model for strain-dependent microtubule activation of Mg-ADP release from kinesin. Nat Struct Mol Biol.

[61] Smiley ST, Reers M, Mottola-Hartshorn C, Lin M, Chen A, Smith TW (1991). Intracellular heterogeneity in mitochondrial membrane potentials revealed by a J-aggregate-forming lipophilic cation JC-1. Proc Natl Acad Sci U S A.

[62] Slutsky I, Abumaria N, Wu LJ, Huang C, Zhang L, Li B (2010). Enhancement of learning and memory by elevating brain magnesium. Neuron.

[63] Branco T, Staras K, Darcy KJ, Goda Y (2008). Local dendritic activity sets release probability at hippocampal synapses. Neuron.

[64] Kaeser PS, Regehr WG (2014). Molecular mechanisms for synchronous, asynchronous, and spontaneous neurotransmitter release. Annu Rev Phys Chem.

[65] Attwell D, Laughlin SB (2001). An energy budget for signaling in the grey matter of the brain. J Cerebr Blood F Met.

[66] Lennie P (2003). The cost of cortical computation. Curr Biol.

[67] Levy WB, Baxter RA (1996). Energy efficient neural codes. Neural Comput.

[68] Nowak L, Bregestovski P, Ascher P, Herbet A, Prochiantz A (1984). Magnesium gates glutamate-activated channels in mouse central neurones. Nature.

[69] Nicholls DG, Ward MW (2000). Mitochondrial membrane potential and neuronal glutamate excitotoxicity: mortality and millivolts. Trends Neurosci.

[70] Miller KE, Sheetz MP (2004). Axonal mitochondrial transport and potential are correlated. J Cell Sci.

[71] Li W, Yu J, Liu Y, Huang X, Abumaria N, Zhu Y (2014). Elevation of brain magnesium prevents synaptic loss and reverses cognitive deficits in Alzheimer's disease mouse model. Mol Brain.

[72] Thevenaz P, Ruttimann UE, Unser M (1998). A pyramid approach to subpixel registration based on intensity. IEEE T Image Process.

[73] Sorzano COS, Thevenaz P, Unser M (2005). Elastic registration of biological images using vector-spline regularization. IEEE T Bio-Med Eng.

[74] Szmacinski H, Lakowicz JR (1996). Fluorescence lifetime characterization of magnesium probes: Improvement of Mg(2+) dynamic range and sensitivity using phase-modulation fluorometry. J Fluoresc.

[75] Yang NC, Ho WM, Chen YH, Hu ML (2002). A convenient one-step extraction of cellular ATP using boiling water for the luciferin-luciferase assay of ATP. Anal Biochem.

[76] Micheva KD, Smith SJ (2007). Array tomography: a new tool for imaging the molecular architecture and ultrastructure of neural circuits. Neuron.

