# Erratum to: Regulation of density of functional presynaptic terminals by local energy supply

**DOI:** 10.1186/s13041-015-0136-8

**Published:** 2015-08-11

**Authors:** Hang Zhou, Guosong Liu

**Affiliations:** Department of Basic Medical Sciences, School of Medicine, Tsinghua University, Beijing, China

## Erratum

After publication of this work [1], it was noticed some of the mathematical equations included on pages 16, 17 and 19 were incorrectly published.

From page 16 “By approximate numerical integration, we derived…” to page 17 “Specifically for Fig. 2b, each data point represented the…”, the correct text is as follows:

By approximate numerical integration, we derived:

$$ {\left[M{g}^{2+}\right]}_i\propto \frac{4}{\pi {\theta}^2d}\cdot \frac{{\displaystyle \sum_{z=-\frac{1}{2}n\rho}^{\frac{1}{2}n\rho }{\displaystyle \sum_{y=0}^{m\theta }{\displaystyle \sum_{x=0}^{l\theta }F\left(x,y,z\right)}}}}{ml}=\frac{4}{\pi {\theta}^2d}\cdot {\displaystyle \sum_{z=-\frac{1}{2}n\rho}^{\frac{1}{2}n\rho}\overline{F(z)}} $$

Where $\overline{F(z)}$ was the mean fluorescence per pixel in the region of the branch in the image (whose coordinate was *z*) in z-stack (Additional file 1: Figure S1.B).

Since we observed that in the range of [−*nρ/2*, *nρ/2*], $\overline{F(z)}$ exhibited well Gaussian distribution (Additional file 1: Figure S1.C), and the normalized distribution curve was the same among different branches (Additional file 1: Figure S1.C), then we knew *Σ*$\overline{F(z)}$ was linearly proportional to $\overline{F{(z)}_{\max }}$ according to the properties of Gaussian curve, thus:

$$ {\left[M{g}^{2+}\right]}_i\propto \frac{4}{\pi {\theta}^2d}\cdot \overline{F{(z)}_{max}} $$

And we could further simplify this formula as follows:

$$ {\left[M{g}^{2+}\right]}_i\propto \frac{\overline{F{(z)}_{max}}}{d} $$

Where $\overline{F{(z)}_{\max }}$ was the mean intensity per pixel in the area of a branch in the compressed image of the stack (the compressed image was achieve by maximal z-projection of the stack, as described above).

From the formula, we knew that [Mg^2+^]_i_ was approximately positively proportional to $\overline{F{(z)}_{\max }}$ but negatively proportional to the diameter of branch, consistent with the experimental observations (Additional file 1: Figure S1.D and E).

In the experiments, given a randomly selected AOI of dendritic area (the image was obtained as described above), 50–100 branches were randomly selected from each AOI and the mean intensity of fluorescence (i.e. $\overline{F{(z)}_{\max }}$) of each branch was measured. Meanwhile, the diameter of each branch (*d*) was measured from the DIC image, then the $\overline{F{(z)}_{\max }}/d$ was calculated to represent the [Mg^2+^]_i_ in the branch. The mean value of these 50–100 branches was calculated to represent the level of [Mg^2+^]_i_ in the AOI. For each coverslip, several AOIs were measured and the mean value of these AOIs was calculated to represent the average level of [Mg^2+^]_i_ in the coverslip. For each data point, several coverslips were used. For each statistical data point, the mean ± SEM of all coverslips was presented. Specifically for Fig. 2b, each data point represented the [Mg^2+^]_i_ (MgGrn fluo. a.u.) value in an individual branch.

On page 19, the correct Additional file 1: Figure S1 legend is as follows:

Additional file 1: Figure S1. Modeling the measurement of [Mg^2+^]_i_ in a segment of branch by MgGrn fluorescence. (A) A segment of branch was modeled as a cylinder characterized by diameter (*d*) and length (*L*). (B) Z-stack of MgGrn fluorescent images was taken in the range of –*h* to *h* at z-axis. $\overline{F(z)}$ represented the mean fluorescent intensity per pixel within a branch area from each layer in the z-stack (left). Theoretically, $\overline{F(z)}$ should exhibited a Gaussian distribution along z-axis (right). (C) Normalized $\overline{F(z)}$ values (normalized to maximum) from z-stacks of 5 representative branches (No.1-5) exhibited well Gaussian distributions with almost the same shape in parallel experiments (Gaussian curve fitting). (D) In the maximal z-projection of the stack, the mean intensity of individual branches $\left(\overline{F{(z)}_{\max }}\right)$ showed a linear correlation with diameter (*d*). (E) After correction, the value $\overline{F{(z)}_{\max }}/d$ was not correlated with diameter in different branches.

In addition to the incorrect equations, it was noted on page 20, reference 39 [2] was also presented incorrectly. The correct reference is included below and in the reference list:

Futai K, Kim MJ, Hashikawa T, Scheiffele P, Sheng M, Hayashi Y. Retrograde modulation of presynaptic release probability through signaling mediated by PSD-95-neuroligin. **Nat Neurosci**. 2007;10(2):186–95. doi: 10.1038/nn1837.
