# Supplementary figures and images for: Regulation of density of functional presynaptic terminals by local energy supply
Source: Mol Brain. 2015 Jul 17;8:42. doi: 10.1186/s13041-015-0132-z (PMC4504454; doi:10.1186/s13041-015-0132-z)

**A**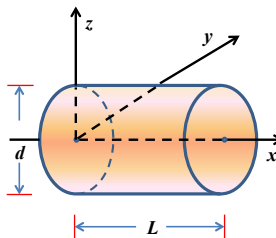**B**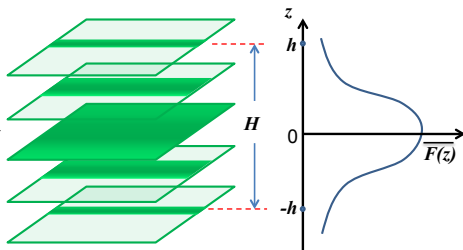**D**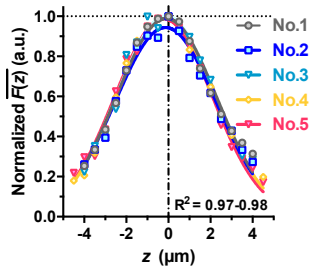**E**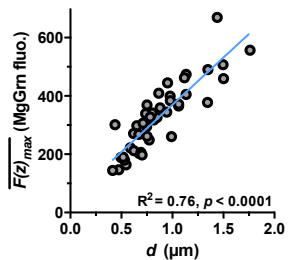**F**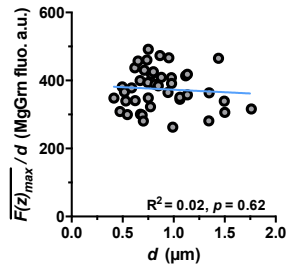

Supplement: Additional file 1: Figure S1. — Modeling the measurement of [Mg2+]i in a segment of branch by MgGrn fluorescence. (A) A segment of branch was modeled as a cylinder characterized by diameter (d) and length (L). (B) Z-stack of MgGrn fluorescent images was taken in the range of –h to h at z-axis. $\frac{\kern0.1em }{F(z)}$F(z) represented the mean fluorescent intensity per pixel within a branch area from each layer in the z-stack (left). Theoretically, $\frac{\kern0.1em }{F(z)}$F(z) should exhibited a Gaussian distribution along z-axis (right). (C) Normalized $\frac{\kern0.1em }{F(z)}$F(z) values (normalized to maximum) from z-stacks of 5 representative branches (No.1-5) exhibited well Gaussian distributions with almost the same shape in parallel experiments (Gaussian curve fitting). (D) In the maximal z-projection of the stack, the mean intensity of individual branches ($\frac{\kern0.1em }{F{(z)}_{\max }}$F(z)max) showed a linear correlation with diameter (d). (E) After correction, the value [file 13041_2015_132_MOESM1_ESM.pdf]
